# Weeds and agro by-products for sustainable farming of edible field cricket, *Gryllus madagascarensis* (Orthoptera: Gryllidae)

**DOI:** 10.1371/journal.pone.0313083

**Published:** 2025-01-03

**Authors:** Henlay J. O. Magara, Cédrique L. Solofondranohatra, Sylvain Hugel, Brian L. Fisher

**Affiliations:** 1 Department of Feed Development, Madagascar Biodiversity Center, Antananarivo, Madagascar; 2 Institut des Neurosciences Cellulaires et Intégratives, UPR 3212 CNRS-Université de Strasbourg, Strasbourg, France; 3 Department of Entomology, California Academy of Sciences, San Francisco, San Francisco, CA, United States of America; National Institute of Agricultural Research - INRA, MOROCCO

## Abstract

*Gryllus madagascarensis* (Orthoptera: Gryllidae) is a cricket species that shows promise to mitigate food insecurity and malnutrition. But whether this species will accept low- to no-cost weeds and agro by-products as feed, and how these feeds affect its performance, remains unknown. This study assessed the acceptability of 66 weed species and agro by-products (derived from a single plant species) by adult *G*. *madagascarensis* and compared the results to a reference feed (chicken feed). We further examined how the 11 top acceptable single plant products affected growth parameters of *G*. *madagascarensis*. The parameters assessed included development, survivorship, body mass and body length and reproductive fitness of the crickets on each of these diets. Finally, the costs of the 11 top accepted single plant products were compared. Our results demonstrated that the cricket accepted all 66 single plant products at varying degrees. Tropical white morning glory (*Ipomoea alba*), cassava tops (*Manhot esculentum*), taro leaves (*Colocasia esculenta*), cowpea bran (*Vigna unguiculata*), American hog-peanut (*Afroamphica africana*), gallant soldier (*Galinsoga parviflora*), wheat bran (*Triticum aestivum*), glycine (*Neonotonia wightii*), silver leaf *Desmodium* (*Desmodium uncinatum*), maize bran (*Zea mays*) and rice bran (*Oryza sativa*) were the most accepted. The analysed nutrient content varied across the top 11 accepted single plant products and the reference feed. The shortest development and highest survival rate were recorded with gallant soldier and cowpea bran powders. Wet body mass and body length were highly impacted by various single plant products tested compared to the reference feed. Reproductive parameters were significantly briefer on tropical white morning glory compared to other feeds and the reference diet. Single plant products cost two- to four-fold less than reference feed. The findings are valuable for developing blended diets that balance performance, cost and availability for household and commercial production of crickets as a “green” technology for producing edible sources of protein.

## 1. Introduction

Rapid increases in the human population, climate change, lack of arable land and destruction of biodiversity threaten food security and nutrition, particularly in sub-Saharan Africa (SSA) [1,2]. The need to feed increasing numbers of people and reverse the effects of climate change and biodiversity loss has sparked a quest for a reliable food supply to support a growing human population. Ideally, alternative food sources should be adaptable to climate change, require little space for cultivation, and be environmentally friendly [3]. Edible insects are a promising solution to these challenges. In the past few years, over 2000 insects have been recorded as alternative food sources for humans around the world [4–7]. In SSA alone 560 insect species are consumed as food [8]. One insect group highly recommended for human consumption is cricket (both Gryllidae and other families of crickets). Globally, over 60 species of crickets are reported to be consumed as food by humans [9]. Of this number, 26 species are eaten in SSA, 41 species in Asia, five species in the Americas and four species each in Europe and Australia [9–11]. Both the nymph and adult stages of crickets are consumed as food [12].

Edible crickets are said to be climate resilient and economical to produce because their rearing requires little space and water and produces neither hazardous nor greenhouse gases [13–15]. They can be reared year-round on locally available diets [16–19]. Edible crickets have also been reported to reach a harvestable stage for consumption earlier than livestock and food crops [20–22]. Further, different studies have documented that eating crickets can benefit human health in several ways. For instance, the protein content of crickets is high, ranging from 35 to 70 g/100g dry matter. The nutritional quality of cricket proteins is considered particularly valuable owing to their amino acid scores and their high digestibility [23,24]. Crickets offer sufficient amounts of all essential amino acids and can provide the lysine, methionine and threonine missing from the edible cereals and legumes consumed by many people in SSA [25,26]. These insects are also rich in mineral elements such as zinc and iron, which are required for proper development [27,28]. Nevertheless, crickets consumed in many biodiversity hot spots are harvested from the wild, making the supply of protein from this source unsustainable and sporadic [29]. This has motivated people to farm edible crickets to help meet the needs for protein in SSA countries such as Madagascar.

Madagascar is an island country in the Indian Ocean with 29.5 million people [30]. Three-quarters of this population is food insecure [31]. This situation has been worsened by drastic climate change, poverty, cyclones, the COVID-19 pandemic, pests, destruction of biodiversity, droughts, poor soils, and lack of arable land as land is owned communally [31–33]. The pervasiveness of malnutrition in Madagascar is high. For example, it is reported that 48% of the children in Madagascar suffer from either chronic (42%) or acute (6%) malnutrition [34–36]. To save these vulnerable children, an intervention is urgently needed to produce protein that is cheap, accessible and sustainable. Most recently, native crickets have been seen as a feasible source of food and protein for Malagasy people. Even resource-constrained people should be able to afford to purchase crickets, unlike more expensive traditional sources of protein [37,38]. The increased relevance of crickets as food in Madagascar has led to an increase in the number of organizations offering crickets for human consumption, spurred research on edible crickets, and triggered a rise in their social acceptance as food [38–40]

Farming native edible crickets such as *Gryllus madagascarensis* in Madagascar can address food insecurity and malnutrition, reduce poverty, conserve biodiversity and improve dwindling soil fertility [3,37,41,42]. However, the main challenge facing cricket farmers in Madagascar is the lack of cricket feed. Identification of a cheap, accessible and sustainable feed for farmed edible crickets remains a subject of extensive research globally [43]. Feed is a key environmental factor influencing cricket population growth [18,44]. To make their cricket product affordable, farmers require cheap, accessible and sustainable feeds [45,46]. Previously, cricket farmers have used high-protein foods such as chicken feed to rear crickets, but these feeds are usually expensive and unsustainable [44]. Other cricket species such as *Acheta domesticus*, *Scapsipedus icipe*, *Modicogryllus conspersus* and *Gryllus bimaculatus* have been reared successfully on diets comprised of by-products of food manufacturing and agriculture [47–50]. In Madagascar, cricket farmers have been using chicken feed to farm edible *G*. *madagascarensis* [40]. However, no study has examined how acceptable weeds and agro by-products available in central Madagascar are to *G*. *madagascarensis*. Moreover, no work has determined whether weeds and agro by-product powders could provide adequate nutrients for the development, survival, growth and reproduction of *G*. *madagascarensis*. This study bridges that information gap by testing weeds and agro by-products available in central Madagascar on their acceptability by *G*. *madagascarensis*. Further, the study compares the development, survival, growth and reproduction of crickets fed on powders made from locally available weeds and agro by-products, and the cost of each weed and agro by-product compared to chicken feed. Finally, we discuss the implications of our findings in light of the effective mass production of *G*. *madagascarensis*.

## 2 Materials and methods

### 2.1 Study site

Collections of weeds and agro by-products were collected in central Madagascar in and around the capital Antananarivo. The bioassay experiments were conducted at the Cricket Rearing and Containment Laboratory (CRCL) of the Madagascar Biodiversity Center (MBC), Antananarivo (18.9326° S, 47.5254° E; approximately 1280 m above sea level) and the nutrition laboratory of Centre National De Recherches sur l’Environnement, Fiadanana, Madagascar.

### 2.2 Rearing of *Gryllus madagascarensis* colony for experiments

The *G*. *madagascarensis* for this study were obtained from the cricket farm at the Madagascar Biodiversity Center in Antananarivo, Madagascar. The colony was established in 2017 using crickets collected from open fields near Antananarivo, Madagascar. Experimental rearing conditions follow that of [51]. The crickets were reared in well-ventilated plastic cages measuring 45 × 30 × 30 cm that were kept in a cricket rearing laboratory at a temperature of 29°C–31°C, 70%-80% relative humidity, and a photoperiod of 12L:12D. Crickets were supplied with blended reference feed “(see below)” placed in plastic dishes. Uneaten chicken feed was renewed every 3 days. Clean tap drinking water was given *ad libitum* through water-soaked cotton balls, which doubled as an oviposition substrate. Eggs laid less than two hours old were collected by unrolling the cotton balls. After being counted, the new eggs were transferred onto moist cotton wool and placed in a plastic container measuring 22 × 16 × 15 cm with ventilation on the lid of the container. The eggs in the cotton were sprayed daily with water using a hand-held pump sprayer, to maintain sufficient humidity until hatching. The incubation containers were placed on a shelf in the cricket-rearing laboratory and monitored regularly every six hours for nymph hatching. The cricket colony was bred for more than eight generations to build up a stable colony before the start of the experiments. The colony of *G*. *madagascarensis* was replenished three times per year with the F1 generation of a trapped population of crickets from the wild to decrease inbreeding.

### 2.3 Weeds, agro by-products, and blended reference feed

#### 2.3.1 Source of weeds and agro by-products and their processing

A total of 48 weeds and 18 agro by-products to be tested singly were collected from the wild, organic farms and stockists in central Madagascar. A weed is any plant growing in an area where it is not wanted while an agro by-products refer to plant materials obtained from grown crops that are not used to produce food. Overall 66 single-plant product species from thirty-three plant families were tested (Table 1). The weeds were opportunistically collected from the wild and farms where pesticides had not been applied. The agro by-products were obtained from crops grown in organic farms in Antananarivo and processed at MBC before being used for experiments. The owners of the farms granted permission to collect weeds and agro by-products. Every sampling point was geolocated with GPS. The mature leaves and flowers and inflorescent and in some cases, pods with seed samples of both weeds and agro by-products were identified taxonomically and vouchers were kept in the MBC botany laboratory. The weeds and agro by-products were cleaned using tap water, dried in the sun, and ground into a fine powder (single plant products) of a grain size 0.01–0.02 mm in diameter using a grinding machine purchased in Antananarivo, Madagascar. The obtained single plant product powders were then used in the acceptance and biology of the cricket experiments.

**Table 1:** Weeds and agro by-products assessed in this study were collected from central Madagascar. Nutritional analyses were conducted on bolded species.

Family	Scientific name and authority	Common name	Malagasy name	Photo	Native or Exotic	Group	Safe for cricket as feed	Season available
**Acanthaceae**	*Thunbergia grandiflora* Roxb.	Blue sky vine	Tsihitafototra	Plate 1	Exotic	Weed	Edible and medicinal	April-July and October-January

Amaranthaceae	*Achyranthes aspera* L.	Devil’s horsewhip	Vatofosa	Plate 2	Native	Weed	Edible by other insects	April-July and October-January
Amaranthaceae	*Amaranthus spinosus* L.	Spiny amaranth	Anapatsa	Plate 3	Exotic	Weed	Edible	April-July and October-January
Amaranthaceae	*Amaranthus viridis* L.	Slender amaranth	Anapatsa	Plate 4	Exotic	Weed	Edible	April-July and October-January
Anacardiaceae	*Mangifera indica* L.	Mango	Manga	Plate 5	Exotic	Agro product	Edible	Year-round
Araceae	***Colocasia esculenta*** (L.) Schott	**Taro**	**Saonjor** **ano**	Plate 6	Native	Weed	Edible	April-July and October-January
Araceae	*Peltandra virginica* (L.) Schott	Green arrow arum	Tsikafona	Plate 7	Exotic	Weed	Not reported	Year-round
Araceae	*Pontederia crassipes* Mart.	Water hyacinth	Kalanchoe	Plate 8	Native	Weed	Edible. Rich in beta carotene	Year-round
Araceae	*Thaumatophyllum bipinnatifidum* (Schott ex Endl.) Sakur., Calazans & Mayo	Split-leaf philodendron	Mananasin’Andriamapandry	Plate 9	Exotic	Weed	Not reported	April-July and October-January
Araceae	*Typhonodorum lindleyanum* Schott	Water banana	Vihy	Plate 10	Native	Weed	Its fruits edible	Year-round
Asparagaceae	*Dracaena trifasciata* (Prain) Mabb.	Snake plant	Sampan-drofia	Plate 11	Exotic	Weed	Not reported	Year-round
Asparagaceae	*Bidens pilosa* L.	Blackjack	Tsipolotra	Plate 12	Exotic	Weed	Edible	April-July and October-January
Asparagaceae	*Furcraea foetida* (L). Haw	Mauritius hemp	Taretra	Plate 13	Exotic	Weed	Eaten by other insects	Year-round
Asteraceae	***Galinsoga parviflora*** Cav.	**Gallant soldier**	**Ananjazavavy**	Plate 14	Exotic	Weed	Edible	Year-round
Asteraceae	*Montanoa hibiscifolia* Benth	Bush daisy	Marigrity	Plate 15	Exotic	Weed	Eaten by goats	April-July and October-January
Asteraceae	*Rudbeckia laciniata* L.	Cutleaf coneflower	Tanamasoandro	Plate 16	Exotic	Weed	Eaten by goats	April-July and October-January
Asteraceae	*Tithonia diversifolia* (Hemsl.) A.Gray	Mexican sunflower	Tanamasoandro	Plate 17	Exotic	Weed	Eaten by goats	April-July and October-January
Basellaceae	*Anredera cordifolia* (Ten.) Steenis	Madeira-vine	Unknown	Plate 18	Exotic	Agro product	Edible	Year-round
Bignoniaceae	*Phyllarthron bojeranum* DC.	Zahana	Zahana	Plate 19	Native and endemic	Weed	edible	April-July and October-January
Boraginaceae	*Symphytum officinale* L.	Common comfrey	Konsoda	Plate 20	Exotic	Weed	Edible by chickens	Year-round
Cannaceae	*Canna indica* L.	Indian Shot	Voaplera	Plate 21	Exotic	Weed	Edible/ no harmful effect	Year-round
Caricaceae	*Carica papaya* L.	Pawpaw	Papay	Plate 22	Exotic	Agro product	Edible	April-July and October-January
Commelinaceae	*Commelina diffusa* Burm.f.	Climbing Dayflower	Nifin’akanga	Plate 23	Native	Weed	Edible	April-July and October-January
Commelinaceae	*Tradescantia pallida* (Rose) D.R.Hunt	Purple heart wandering jew	Nifin’akanga	Plate 24	Exotic	Weed	Edible	Year-round
Commelinaceae	*Commelina benghalensis L*.	Tropical spiderwort	Nifin’akanga	Plate 25	Native	Weed	Edible	April-July and October-January
Convolvulaceae	***Ipomoea alba*** L.	**Tropical white morning glory**	**Ravimbomanga diha**	Plate 26	Exotic	Weed	Edible	April-July and October-January
Convolvulaceae	*Ipomoea batatas* (L.) Lam.	Sweet potatoes vines	Vomanga	Plate 27	Exotic	Agro product	Edible	April-July and October-January
Convolvulaceae	*Ipomoea indica* (Burm.f.) Merr.	Common morning glory	Ravimbomanga diha	Plate 28	Exotic	Weed	Edible	April-July and October-January
Cucurbitaceae	*Cucurbita maxima* Duches	Pumpkin	Ravim-boatavo	Plate 29	Exotic	Agro product	Edible	April-July and October-January
Cucurbitaceae	*Sicyos edulis* Jacq.	Pear squash	Saosety	Plate 30	Exotic	Agro product	Edible	April-July and October-January
Crassulaceae	*Kalanchoe pinnata* Raym.-Hamet & H.Perrier	Cathedral bells plant	Sodifafana	Plate 31	Exotic	Weed	Edible and medicinal	April-July and October-January
Cyperales	***Oryza sativa*** L.	**Rice**	**Vary**	Plate 32	Exotic	crop	edible	December- May
Cyperales	***Triticum aestivum*** L.	**Wheat**	**Varimbazaha**	Plate 33	Exotic	crop	edible	December- May
Euphorbiaceae	*Euphorbia stenoclada* Baill.	Stiver Thicket	Samanta	Plate 34	Native	Weed	Used as cattle feed	Year-round
Euphorbiaceae	***Manihot esculenta var*. *a*** L.	**Cassava with red stalk**	**Mangahazo**	Plate 35	Exotic	Agro product	Edible	April-July and October-January
Euphorbiaceae	*Manihot esculenta* var. b Crantz	Cassava with a green stalk	Mangahazo	Plate 36	Exotic	Agro product	Edible	April-July and October-January
Fabaceae	***Afroamphica africana*** (Hook. f) H. Ohashi and K. Ohashi	**American hog-peanut**	**Mandalo dia miraikitra**	Plate 37	Exotic	Weed	Edible	April-July and October-January
Fabaceae	***Desmodium uncinatum*** (Jacq.) DC.	**Silverleaf desmodium**	**Mandalo dia miraikitra**	Plate 38	Exotic	Weed	Edible	November-July
Fabaceae	*Desmodium intortum* (Mill.) Urb.	Green leaf desmodium	Mandalo dia miraikitra	Plate 39	Exotic	Weed	Edible	November-July
Fabaceae	***Neonotonia wightii*** (Wight & Arn.) J.A. Lackey	**Glycine**	**Malay**	Plate 40	Exotic	Weed	Edible	Year-round
Fabaceae	*Phaseolus vulgaris*	Navy beans	Tsaramaso fotsy	Plate 41	Exotic	Agro by-product	Edible	Year-round
Fabaceae	*Senna occidentalis* (L.) Link	Coffee senna	Tsotsorinangatra	Plate 42	Exotic	Weed	Edible	April-July and October-January
Fabaceae	***Vigna unguiculata*** (L.) Walp.	**Cowpeas**	**Voanemba**	Plate 43	Exotic	Agro by-product	Edible	Year round
Fabaceae	*Wisteria frutescens* (L.) Poir.	American wisteria	Glycine	Plate 44	Exotic	Weed	Edible	April-July and October-January
Lamiaceae	*Salvia coccinea* Buc’hoz ex Etl.	Red sage	Unknown	Plate 44	Exotic	Weed	Edible	April-July and October-January
Lauraceae	*Persea americana* Mill.	Avocado	Zavoka	Plate 46	Exotic	Agro product	Edible	Year-round
Meliaceae	*Melia azedarach* L.	Chinaberry tree	Voandelaka	Plate 47	Exotic	Weed	Not edible	Year-round
Musaceae	*Musa acuminata* Colla	Banana	Akondro	Plate 48	Exotic	Agro product	Edible	Year-round
Nyctaginaceae	*Bougainvillea glabra* Choisy	Paper flower	Laingomena	Plate 49	Exotic	Weed	Not edible	Year-round
Nymphaeaceae	*Nymphaea nouchali* Burm.f.	Dwarf aquarium lily	Voahirana	Plate 50	Native	Weed	Edible	Year-round
Oleaceae	*Fraxinus ornus* Marshall	Manna ash	Unknown	Plate 51	Exotic	Weed	Edible	April-July and October-January
Oxalidaceae	*Oxalis latifolia* Kunth	Broadleaf wood sorrel	Kididiam-borona	Plate 52	Exotic	Weed	Edible	April-July and October-January
Passifloraceae	*Passiflora subpeltata* Ortega	White passion flower vine	Garana diha	Plate 53	Exotic	Weed	Edible	April-July and October-January
Poaceae	*Bromus catharticus* Vahl	Rescue grass	Bozaka	Plate 54	Exotic	Weed	Edible	April-July and October-January
Poaceae	*Cenchrus clandestinus* (Hochst. Ex Chiov.) Morrone	Kikuyu grass	Bosaka	Plate 55	Native	Weed	Edible	April-July and October-January
Poaceae	*Cynodon dactylon* (L.) Pers.	Bermuda grass	Fandrotrarana	Plate 56	Native	Weed	Edible	April-July and October-January
Poaceae	*Phyllostachys aurea* (André) Rivière & C.Rivière	Golden bamboo	Bararata	Plate 57	Exotic	weed	Edible	Year round
Poaceae	*Saccharum officinarum* L.	Sugarcane	Fary	Plate 58	Exotic	Agro product	Edible	Year-round
Poaceae	***Zea mays*** L.	**Maize or corn**	**Katsaka**	Plate 59	Exotic	Agro product	Edible	April-July and October-January
Saururaceae	*Houttuynia cordata* Thunb.	Chameleon plant	Unknown	Plate 60	Exotic	Weed	Edible in Vietnam and the Far East	April-July and October-January
Solanaceae	*Brugmansia suaveolens* (Humb. & Bonpl. ex Willd.) Sweet	Angel’s trumpet	Seva	Plate 61	Exotic	weed	Eaten by goats	April-July and October-January
Solanaceae	*Solanum Lycopersicum* L.	Tomatoes	Voatabia	Plate 62	Exotic	Agro product	Edible	April-July and October-January
Solanaceae	*Solanum americanum* Mill.	Black nightshade	Anamamy diha	Plate 63	Exotic	Weed/ agro product	It is edible in Africa and other parts of the world	April-July and October-January
Thelypteridaceae	*Christella normalis* Schmidel	Maiden ferns	Unknown	Plate 64	Exotic	Weed	Not reported	April-July and October-January
Thelypteridaceae	*Nephrolepis exatata* (L.) Schott	Dwarf feather palm ferns	Unknown	Plate 65	Exotic	Weed	Not reported	April-July and October-January
Zingiberaceae	*Hedychium coronarium* J. Koenig	White ginger lily	Longozo	Plate 66	Exotic	Weed	Edible	Year-round

#### 2.3.2 Identification of weeds and agro by-products

The samples of different weeds and agro by-products harvested together with their flowers and fruits were pressed between newspapers and put into gunny sacks with labels (locality, farm, name of the collector, date collected). Then weeds and agro by-product samples were transported to the MBC botany laboratory where they were sorted and identified up to the species level using appropriate dichotomous keys [52]. To confirm whether the identification was done correctly, the identified samples were counterchecked against stored herbarium specimens at TAN herbaria (Parc Botanique et Zoologique de Tsimbazaza). The photos were taken on respective samples corrected. The identified specimens were pressed and dried between old newspapers, transferred into specimen boxes and kept as vouchers in the MBC botany laboratory in Antananarivo, Madagascar. The status of each species as native or endemic to Madagascar vs. introduced (exotic) was determined based on known plant distribution records [53].

#### 2.3.3 Reference diet

Chicken feed was used as the reference diet. Chicken feed is a blended diet of five plant products such as maize meal, soya bean meal, rice bran, a mixture of finely milled wheat bran and wheat flour and broken rice. Other ingredients in the chicken feed include lime, dicalcium phosphate, vitamin-mineral premix, table salt, methionine and lysine amino acid. The feed used was produced by LFL Feed Company, Antananarivo, Madagascar.

### 2.4 Assessment of the acceptance of the weeds and agro by-products by *Gryllus madagascarensis*

The acceptability of 48 weeds and 18 agro by-product powders (single plant products) were compared to blended reference feed by *G*. *madagascarensis* in a no-choice test. The criteria for acceptance of the test single plant products were based on the quantity of the single plant product or the reference feed consumed by the crickets. Each acceptability experiment was replicated five times using five males and five and repeated three times leading to n = 30 (15 males and 15 females per feed). Before each experimental trial on the acceptability of single plant products and reference feed, 335 six hours old after emergence to adult males and 335 six hours old after emergence to adult females of *G*. *madagascarensis* were randomly picked from the rearing cage and transferred into two holding cages according to the sex of the cricket. Each holding cage measured 45 (length) × 30 (width) × 30 (height) cm with a ventilation hole on the lid measuring 30 x 23 cm to which wire gauze with a hole size: 0.5 mm was glued. From each holding cage, all the female and male adult crickets were individually transferred into rectangular boxes each measuring 16 x 10 x 7.5 cm for the experiment (n = 30). Each cricket was starved for 24 hours before the start of the experiments. After 24 hours of starvation, 5 boxes containing a single female and another 5 boxes with a single male of *G*. *madagascarensis* were selected for each feed tested in the first round of the experiment. The same boxes were used in the repeat experiments. Each cricket was then provisioned with one gram of test single plant product or reference feed, water in a cotton ball and a small piece of egg crate to hide in [54]. All the crickets in 670 boxes (335 for male crickets and 335 for females) were allowed to feed on single plant products and reference feed for 24 hours. After 24 hours, the mixture of uneaten single plant products or reference feed and cricket dropping was withdrawn from each box. Cricket droppings which are granules in nature were then separated from the feed residue by sieving the mixture of recovered feed and cricket droppings through a sieve of dimensions 1mm by 1mm whereby the cricket droppings (with a diameter of 1.1 mm to 1.2 mm) were trapped in the sieve and the uneaten feed passed down into a glass petri dish of diameter 12 cm. The separated uneaten single plant products (weeds and agro by-products), reference and the cricket droppings were then dried in an oven at 40°C for four hours before weighing [55]. The mass of single plant products and reference feed consumed by individual crickets was obtained by subtracting the dry matter of the unconsumed weed, agro by-product powder and chicken feed from the dry matter of the respective single plant products and chicken feed provided at the start of the experiment [56]. In instances where the cricket died or did not consume feed, the data was excluded from the analysis. The population of the crickets used to study the three runs of acceptability experiment were 1005 male and 1005 female (N = 2010 crickets). All experimental groups were kept in an environmental room with the same controlled conditions at MBC, Madagascar: 30 ± 1°C and 75% ± 5% RH, with a photoperiod of 12L:12D and allowed to feed *ad libitum*.

### 2.5. Nutritional analysis of the top 11 accepted weeds, agro by-products and reference feed

Nutritional profiles of the top 11 preferred single plant products and the reference diet were assessed by the Nutrition Laboratory of Centre National de Recherches sur l’Environnement, Fiadanana, Madagascar. Crude fat, crude protein, crude fibre, and ash contents of the test diets were analyzed using the official methods of the Association of Official Analytic Chemists [57]. The percentage of moisture in each type of feed was determined by drying each diet sample in an oven with a temperature maintained at 110°C for 24 hours. Each dried sample was cooled to room temperature in a desiccator before reweighing using a Mettler P1210 analytical balance to obtain a constant weight. The carbohydrate content was calculated by subtracting the percentage content of crude protein, crude fat, crude fiber and ash from 100% of dry matter [58]. The gross energy value (kcal/kg) was calculated by summing the percentages of proteins and carbohydrates and multiplying the total by forty before adding it to the total lipids multiplied by a factor of ninety [58]. An atomic absorption spectrometer (AAS) was used to determine the mineral composition of each feed [59].

### 2.6. Assessment of the effect of the weeds, agro by-products and reference feed on the development, survival, wet body mass and body length and reproductive parameters of *G*. *madagascarensis*

To investigate the effect of single plant products on the biological parameters of *G*. *madagascarensis*, six weeds and five agro by-products were selected based on their acceptance and abundance across Madagascar, chicken feed served as the reference diet. The single plant product was deemed accepted when the cricket consumed some mass of the feed offered to it and the presence of a live cricket by the time of the stoppage of the experiment. On the other hand, the abundance of the weeds and agro by-products was determined by crosschecking how easily they are available in terms of large quantities in various localities in Madagascar where crickets are grown. The selected weeds included tropical white morning glory powder, gallant soldier powder, taro leaf powder, silverleaf *Desmodium* powder, American hog-peanut powder and glycine leaf powder. The agro by-products picked included cassava leaf tops, cowpea bran, wheat bran, maize bran and rice bran. The parameters of *G*. *madagascarensis* measured included nymph development time, survivorship, wet body length, wet body weight, preoviposition duration, fecundity, egg incubation duration and egg hatchability. All traits measured are defined below in each subtitle.

#### 2.6.1 Effect of weeds, agro by-products and reference feed on nymphal development and survivorship of *G*. *madagascarensis*

The impact of single plant products and blended reference feed on the development time and survivorship were assessed on 100 one-day-old nymphs individually per each feed tested. This means that each single plant product and blended reference feed experiment had 100 replicates and each experiment was repeated three times at different times (n = 300 crickets). During the feeding trial, each nymph of *G*. *madagascarensis* was then offered 0.5 g of particular single plant products or blended reference feed served on a plastic lid with a diameter of 4 cm and followed until the adult stage. The experimental cages were cleaned every three days, during which the diet was replenished and water-soaked cotton balls changed. Additionally, each cricket was offered a piece of egg crate as a hideout. Single plant products and the reference for replenishing were carried out on the second day using a digital weighing balance and kept in trays covered with other trays to prevent the feeds from absorbing moisture. This was aimed to reduce the workload on the third day of replacing the uneaten feed with the fresh one. The fresh cotton balls for supplying water to the crickets were also prepared on the second day awaiting exchange. During the replenishment of the feed and water, the cricket was scared by tapping the container with a finger to make it hide in the piece of egg crate. Then the hiding cricket was transferred into another small container to allow cleaning and replenishing of feed and water. Afterwards, the cricket is returned to the cleaned cage with fresh feed and water. The trained research assistant assisted in exchanging feed and water. The insects in the cages were monitored daily as they developed. Any mortality that occurred was recorded. Nymphal development is the time between the hatching and eclosion of adult crickets [60]. The per cent survival rate was obtained by determining the number of individuals alive at the end of the experiment divided by the initial number multiplied by 100 [61].

#### 2.6.2 Effect of weeds, agro by-products and reference feed on wet body mass and body length of *G*. *madagascarensis*

To determine the wet body mass and body length of male and female *G*. *madagascarensis*, 12 males and 12 females newly molted adults from the last juvenile fed on each single plant product were randomly picked and introduced individually into a transparent plastic cup (7.5 cm diameter × 12.5 cm height) covered with a transparent petri dish. Each experiment was repeated three times (n = 72). Into each plastic cup, a 6-hour-old adult cricket after emergence from the last juvenile was introduced and the opening of the cup was covered with a transparent petri dish to restrain the cricket from not jumping out. Then the cricket in the cup was allowed 3–5 minutes to settle down, and then its length was measured by placing a ruler under the container parallel to the cricket. The lower limit of measurement by the ruler was 1mm. The wet body mass was also recorded using a digital electronic weighing machine with 0.0001 g readability (Kern and Sohn, Ballngen, Germany). The cricket in the cup hardly moved when weighed. We recorded the body mass for each cricket when the reading on the balance screen did not change anymore. The cricket body mass of the cricket was recorded as the total mass of the cup containing the cricket minus the transparent cup mass [60]. The sex of the crickets was determined by checking the presence of an ovipositor in females [62].

#### 2.6.3 Effect of weeds, agro by-products and reference feed on the reproduction performance of *G*. *madagascarensis*

To assess the effects of single plant products and blended reference feed on reproductive parameters, 12 pairs of newly moulted adult males and females (six hours old) of *G*. *madagascarensis* with wings covering the whole body were subjected to each of the experimental diets and followed daily until they died. The 144 pairs used for the experiment in one round were easily obtained at nearly the same time since the eggs to run the test experiments were collected within two hours. This meant the eggs hatched at the same time, leading to adult crickets also emerging at the same time. Then each pair was placed in a well-ventilated transparent plastic cage measuring 22 × 16 × 15 cm (1200 ML Rectangle Super—2; Aristo Manufacturers Limited, India) and randomly placed on shelves in the experiment room. The paired crickets were maintained under the above-mentioned conditions and feeding regimes. After cleaning and food replenishment, the position of containers was interchanged randomly to avoid the possibility of location influence [63]. Each cage with male and female crickets was offered a moist cotton ball (70% moist) at 6 p.m. daily which served as an oviposition medium. The female crickets were allowed to lay eggs for 24 hours (6.00 pm to 6.00 pm) equivalent to a day until the time female crickets died. The cotton balls were then withdrawn after 24 hours and replaced with fresh ones. The cotton ball for oviposition were replaced daily throughout the lifetime of adult female crickets. When the male cricket died before the female cricket, another male cricket was picked from the mother colony to replace it. But if the female cricket died, the collection of eggs from that cage was terminated. To determine whether the female cricket had laid eggs, the presence of eggs was checked on withdrawn cotton by opening the balls and separating the balls into thin cotton sheets. The cotton ball did not have eggs in the first few days marking the preoviposition period. Preoviposition time was obtained from the emergence of females to the first day of egg laying [45]. It varied in different single plant products fed on by the pair of crickets. When female crickets started laying, the eggs could be seen deposited in the cotton balls. Each female’s oviposited eggs were counted and incubated in 1000 ml rectangular plastic cages measuring 16 × 10 × 7.5 cm (Super—2; Aristo Manufacturers Limited, India), ventilated with 0.2 mm gauge wire mesh on the lid at the top. The eggs were put on a wet sheet of cotton wool and then covered with another sheet of cotton. The cotton wool was sprinkled with water every morning to prevent the eggs from drying out. The egg containers were checked after six hours daily for newly hatched nymphs. Nymph numbers were recorded until hatching stopped completely. Fecundity was calculated as the sum of the number of eggs deposited by each female in a period of 24 hours until the female cricket dies [60]. Time until hatching was determined from the day the egg was laid until hatching occurred [24]. The proportion of the eggs that hatched (egg hatchability) was calculated as the number of hatched eggs divided by the total number of eggs oviposited by each female cricket multiplied by 100% [64]. This was achieved by observing the progress of egg hatching while removing egg shells from the container with eggs into an empty container using a fine brush. The removed egg shells were counted and the number was recorded. The egg shells here represented the number of hatched eggs [45]. The time taken by the eggs to hatch was calculated from the onset of hatching to the time the eggs stopped hatching completely.

### 2.7 Cost of the selected weeds and agro by-products powders and reference feed, initial body mass, average daily gain, feed consumed by one kilogram of crickets, the feed conversion ratio and the feed cost per body weight gain

The single weeds, such as tropical white morning glory, gallant soldier, silver leaves *Desmodium*, glycine, and American hog-peanut (but excluding taro leaves) used in the study of the biology of the cricket did not incur any purchasing costs since we harvested them freely from the farms after seeking permission from landowners. Cassava leaf tops, an agro by-product, and taro leaves (a weed) were purchased from organic farms. Wheat bran, rice bran, cow pea bran and maize bran agro by-products were purchased from a stockist by the kilogram. The cost of one kilogram of agro by-products and taro leaves was based on the price farmers or stockists charged us. The cost of one kilogram of the blended reference diet was obtained from the stockist of reference feed in Antananarivo.

To assess the impact of the single plant products and the reference on feed conversion ratio (FCR): 1200 nymphs were divided into 12 groups (100 nymphs per group) and were kept individually per cage. They were randomly introduced to the 11 single plant product and the reference feed. Each cricket per cage was provided with enough feed (0.5 g of each feed treatment) and enough water. All the 100 individual crickets fed a single plant product or reference feed were weighed separately every seven days as reported by [14]. The amount of a single plant product or reference feed consumed by an individual cricket per group was weighed twice per week as the feed was exchanged after three days until the time of adult emergence. To determine daily feed intake (kg), it was calculated by recording the quantity of a single plant product or reference feed consumed (g) each week and dividing it by seven days. Average daily mass gain was obtained by subtracting the initial body mass of the cricket from the final body mass gained since the last weighing divided by the time taken by time taken by the nymphs to reach the adult stage per feed. The FCR was determined by dividing the daily feed intake/average mass gained by the cricket. The number of crickets needed to produce one kilogram of live weight gain per single plant product and reference feed was calculated by dividing 1000 g equivalent to one kilogram of crickets by the final mass gain per cricket. The consumed amount of feed (kgDM) per kg live weight gain was determined by multiplying the number of crickets making one kilogram per feed with the feed consumed by each cricket to the adult stage and dividing by 1000. The cost of feeding crickets of single plant product and reference feed was also obtained as the cost of feeding the crickets per kg live weight gain = cost per kg of single plant product or reference feed × feed consumed by one kilogram of crickets per feed. The average cost of feeding per kg live weight gain was then calculated. The first feeding experiment was conducted from October to December 2022, the second feeding experiment from January to march 2023, and the last feeding experiment from April to June 2023.

### 2.8 Statistical analysis

A one-way analysis of variance (ANOVA) was used to test whether acceptance of the single plant products and blended reference feed differed. The variations in the survival rate of *G*. *madagascarensis* nymphs to adults and the percentage hatchability of eggs between different single plant products and reference feed were analysed using a logit-linked binomial generalised linear model (GLM). Differences in pre-oviposition time, fecundity and duration of egg incubation among different single plant products and blended reference feed were determined using a negative binomial GLM, which was used to correct over-dispersion that occurred when the data were distributed according to a log-linked Poisson distribution. Over-dispersion resulted when the female crickets failed to lay eggs for some days. One-way analysis of variance (ANOVA) was used to analyse data on development time, body weight and body length. Tukey’s honest significant difference (HSD), a post-hoc test, was used as a multiple-comparison procedure to assess where categories differed significantly. A pairwise Wilcoxon test was used to calculate pairwise comparisons between group levels. Pearson correlation coefficient (r) was tested to determine whether there was a linear correlation between body mass and fecundity and body length and fecundity. All effects were considered significant at p < 0.05. All analyses were performed using R version 4.3.1 [65]. statistical software.

## 3 Results

### 3.1 Sourced and identified weeds and agro by-products

**Fig 1:**
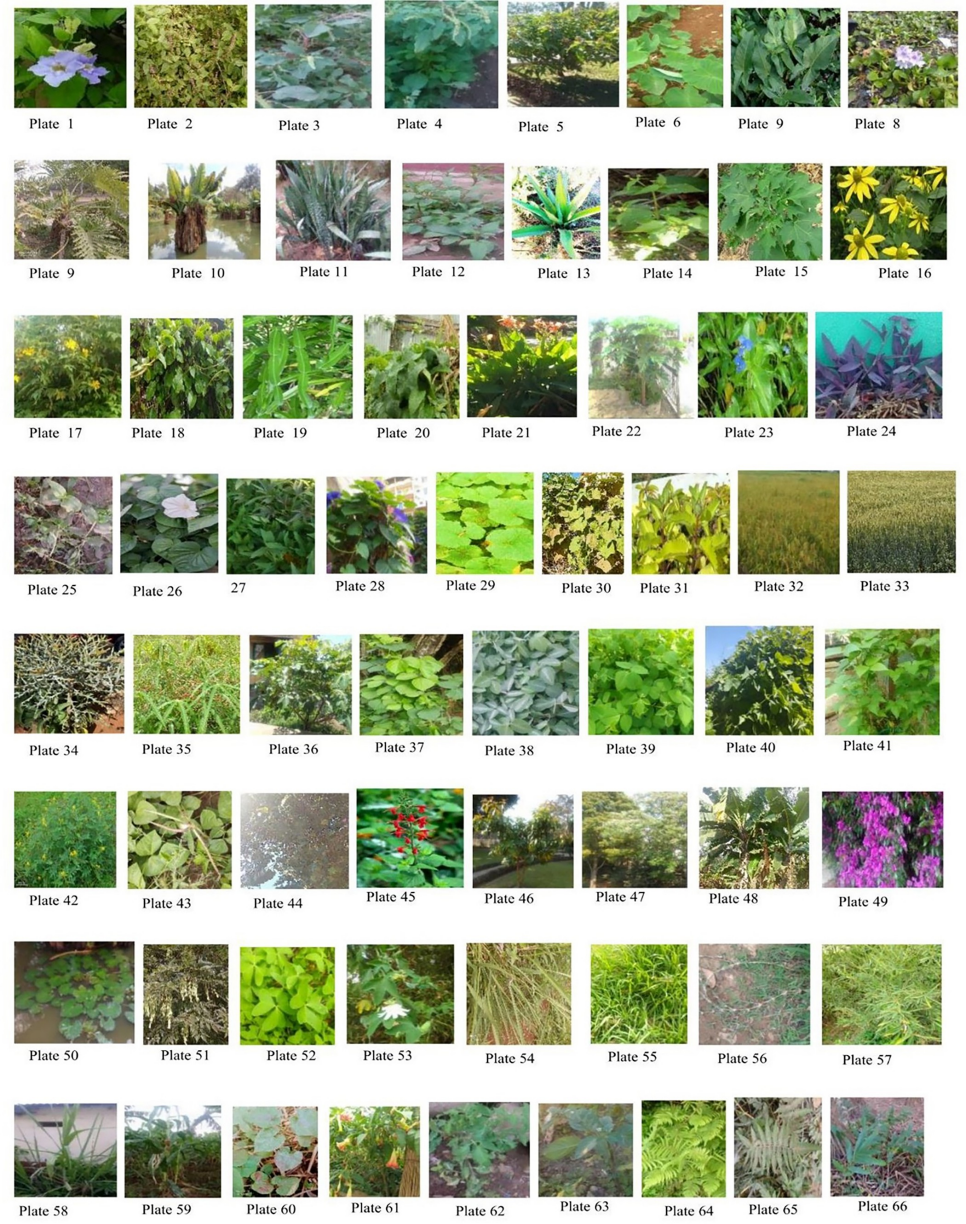
Photos of various weeds and agro by-products from central Madagascar used for the study.

Our first objective was to source available agro by-products and to source and identify weeds available in central Madagascar. During the study period, a total of 48 weeds and 18 agro by-product samples belonging to 66 species from thirty-three families were gathered and identified at the species level (**Table 1 and Fig 1**).

The weeds and agro by-products selected for trial feeding are indicated in bold. Weeds selected included taro (*Colocasia esculenta*), gallant soldier (*Galinsoga parviflora*), tropical white morning glory (*Ipomoea alba*), American hog-peanut (*Afroamphica africana*), silverleaf desmodium (*Desmodium uncinatum*) and glycine (*Neonotonia wightii)*. Agro by-products included rice (*Oryza sativa*), wheat bran (*Triticum aestivum*), cassava with red stalks (*Manihot esculenta)*, cowpeas (*Vigna unguiculata*), and maize or corn (*Zea mays*).

### 3.2 Acceptance of weeds and agro by-products and reference feed by adults of *G*. *madagascarensis*

**Fig 2:**
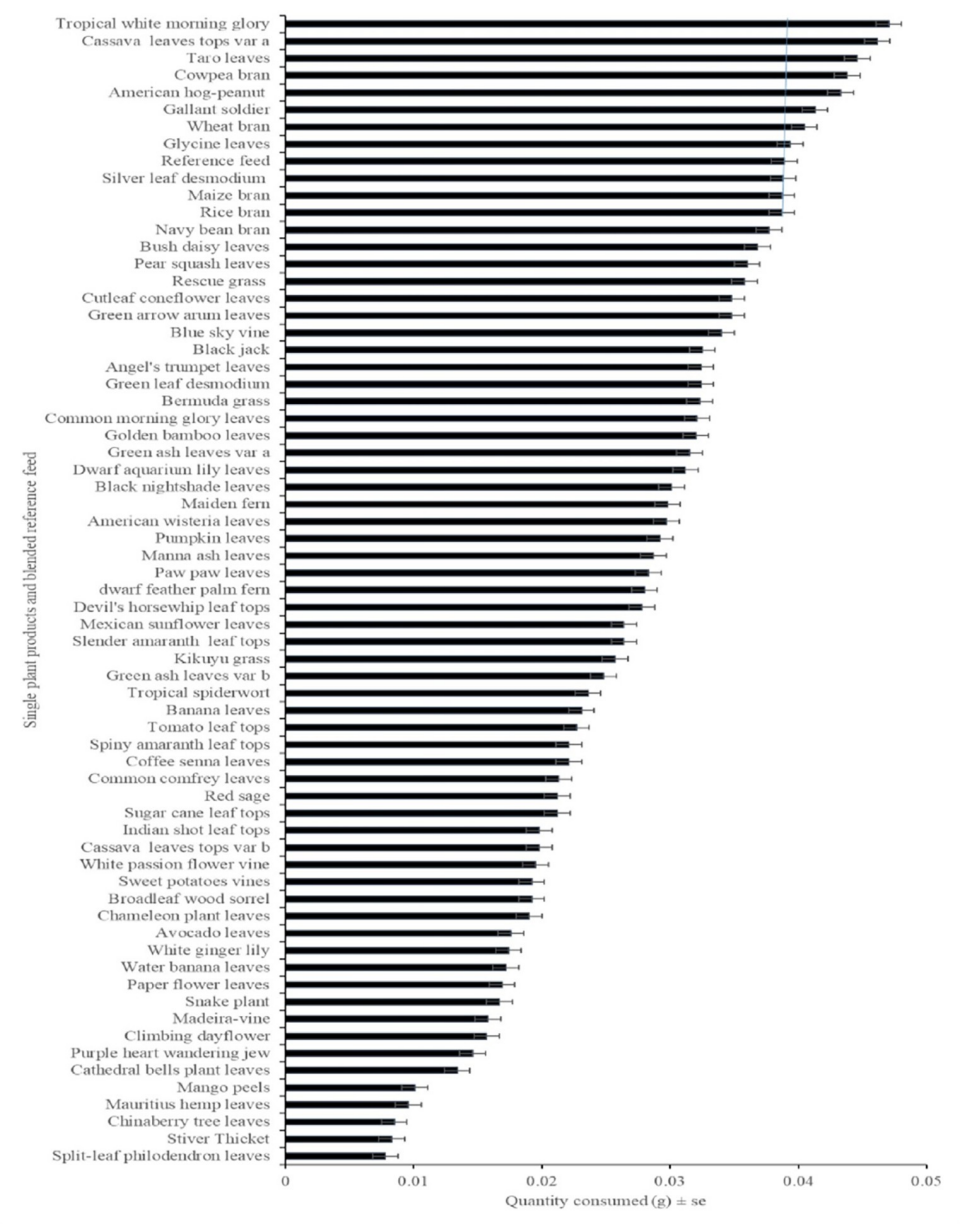
Acceptance of the 66 single plant products and blended reference feed based on no-choice test (n = 30). The histogram shows the mean ± se quantity of single plant products compared to the reference diet, blended reference feed consumed by *G*. *madagascarensis* adult males and females in 24 hours. The tested feeds are ordered by the amount consumed. The blue line shows the value for chicken feed, used as a reference for single plant products.

A prerequisite for a feed to be usable is its acceptance by the farmed cricket species. In the no-choice test, the 48 weeds and 18 agro by-products (single plant products) and blended reference feed tested were fed to adults of *G*. *madagascarensis*. All tested single plant products were accepted to some extent, but acceptance differed significantly between them, with the most accepted single plant products three to four times more consumed than the least accepted (F_66, 1943_ = 60.97, p < 0.001) (**Fig 2**). Overall, tropical white morning glory powder, cassava tops powder, taro leaves powder, cowpea bran, American hog-peanut powder, gallant soldier powder, wheat bran, glycine powder, chicken feed, silver leaf *Desmodium* powder, maize bran and rice bran were the most accepted (**Fig 2 and S1 Fig in S1 File**). Most of these accepted single plant products are widely distributed in Madagascar. We will therefore focus our assessment on these five agro-by products and the six most accepted weeds. In addition, our acceptance data also revealed a strong interaction between the single plant products tested and the sex of the cricket (F_66, 1943_ = 2.474, p = 0.0085). Both females and males of *G*. *madagascarensis* showed a significant variation in acceptance of the single plant products and reference feed offered to them (male adult F_1, 1943_ = 53.68, p < 0.001; female adult F_1, 1943_ = 38.12, p < 0.001) (**S1 Fig in S1 File**).

### 3.3 Nutritional composition of the selected single weeds and agro by-products and blended reference feed

In addition to acceptance by crickets, the nutritional composition of single plant products is a critical parameter to be considered when selecting a feed. We therefore analyzed the nutritional composition of the 11 most accepted weeds and agro by-products powders and blended reference feed (**Table 2)**. One of the most important parameters for a feed is the amount of protein, carbohydrate and lipid it contains. The quantity of crude protein in cassava leaf tops (25.19% protein) and tropical morning glory (23.48% protein) was higher than that of other single plant products and the reference feed (20.03% protein) used in the experiment. The lipid composition of silver leaf *Desmodium* leaves (1.33% lipid) was the lowest while the highest was for maize bran (9.65% lipid). The gallant soldier had the highest crude fiber (29.30% fiber) while the reference feed had the least (0.30% fiber). The silverleaf *Desmodium* had the highest carbohydrate content (68.82% carbohydrates) compared to the rest of the plant products and the reference feed. The maize bran contained the most gross energy (3688.90 kcal/kg DM). The content of metals is important, not only for the insect but also for the final destination of the farmed insects (i.e. human consumption). Cowpea bran had the highest levels of zinc (57.90 mg/kg) compared to single plant products. Cassava leaf powder contained the highest iron (6.15 mg/kg) compared to other single plant products. The highest quantity of calcium (29.50 mg/kg) and magnesium (6.10 mg/kg) was recorded in wheat bran and the least calcium and magnesium were recorded in maize bran (0.10 mg/kg) and silverleaf *Desmodium* (1.30 mg/kg) respectively. The highest level of sodium was associated with rice bran (2.90 mg/kg) and the lowest level of sodium was recorded in maize bran (0.01 mg/kg). Taro leaves contained the highest quantities of potassium (40.20 mg/kg) and manganese (523.80 mg/kg) compared to other feeds. The copper content of blended reference feed (14.00 mg/kg) and tropical white morning glory (12.60 mg/kg) were higher than that of the other feed types. Importantly, no lead was detected in any of the feed types. In addition, the ash content of rice bran and gallant soldier was higher than that of other experimental single plant products and the reference feed.

**Table 2:** Nutritional composition (DM basis) of single plant products and blended reference feed tested for rearing *G*. *madagascarensis*.

Nutrition composition	Blended reference feed (Chicken feed) (E)	Weeds						Agro by-products				
		Silver leaf *Desmodium* (MM)	Gallant soldier (GS)	Taro leaves (TD)	Tropical white morning glory (IA)	Glycine (GW)	American hog-peanut (HN)	Cassava leaves (C)	Cowpeas bran (CP)	Wheat bran (WB)	Maize bran (MB)	Rice bran (R)
Moisture %	10.35	8.15	9.77	8.04	7.43	7.50	7.80	8.24	8.50	8.04	11.44	9.10
Ash %	8.27	8.50	15.40	12.58	12.13	6.70	10.6	8.83	3.50	5.34	3.95	15.45
Crude protein %	20.03	13.20	19.03	23.13	23.48	20.5	14.80	25.19	22.50	14.40	11.02	6.71
Lipid %	4.04	1.33	3.03	4.00	2.16	3.10	1.34	4.19	1.70	4.25	9.65	5.78
Crude fiber %	0.30	25.50	29.30	22.2	19.8	13.6	21.9	17.30	0.80	6.54	20.70	7.50
Carbohydrate %	57.31	68.82	52.77	52.25	54.80	52.93	65.91	53.55	58.34	64.52	63.94	62.96
Gross energy (kcal/kg DM)	3457.20	3400.50	3144.70	3375.20	3325.60	3216.20	3349.00	3526.70	3386.60	3539.30	3866.90	3307.00
Zinc (mg/kg)	13.10	31.60	46.70	25.70	26.40	46.70	33.20	49.60	62.02	57.90	61.70	36.60
Iron (mg/kg)	6.50	2.88	5.30	2.42	2.33	2.68	2.64	6.15	4.68	4.62	1.56	0.11
Calcium (mg/kg)	16.40	10.40	21.90	13.00	23.60	7.40	11.90	13.20	7.30	29.50	0.10	2.20
Magnesium (mg/kg)	1.70	1.30	3.70	2.90	2.40	5.29	2.30	1.60	1.40	6.10	2.00	2.10
Sodium (g/kg)	1.50	0.10	0.30	0.10	0.10	0.81	0.16	0.10	0.12	2.13	0.01	2.90
Phosphorus (mg/kg)	4.20	1.30	4.50	2.70	3.90	1.78	2.40	2.60	1.10	9.50	2.90	2.70
Potassium (g/kg)	7.90	13.60	38.20	40.20	22.40	12.00	19.90	13.40	24.50	12.17	6.60	7.10
Manganase (mg/kg)	13.10	57.30	100.40	523.80	62.50	52.90	33.20	73.90	101.00	76.90	25.20	141.40
Copper (mg/kg)	14.00	6.10	8.10	4.70	12.60	8.70	7.30	5.30	4.60	5.40	4.50	4.60
Lead (mg/kg)	nd	nd	nd	Nd	nd	nd	nd	nd	nd	nd	nd	nd

### 3.4 Effect of weeds, agro by-products and reference feed on developmental time, survivorship, wet body mass, body length, and reproductive parameters of *G*. *madagascarensis*

#### 3.4.1 Effect of single weeds and agro by-products powders and blended reference feed on the developmental time of *G*. *madagascarensis*

**Fig 3:**
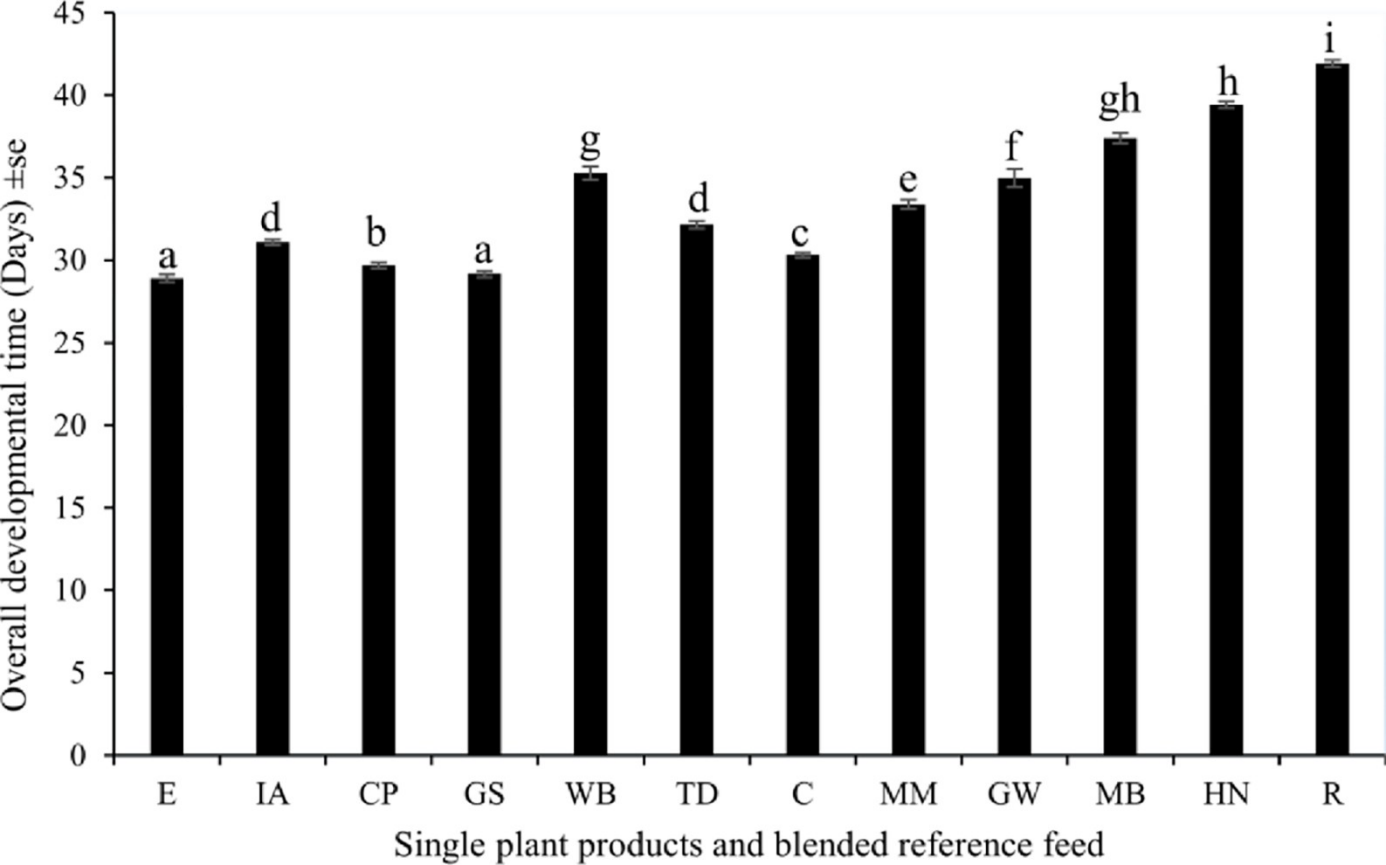
Mean (± se) development time of *G*. *madagascarensis* reared on different weeds and agro by-products and chicken feed (n = 300). E- Blended reference feed, C-Cassava leaves powder, CP-Cowpeas powder, MM-Silverleaf *Desmodium* leaf powder, GS-Gallant soldier powder, MB-Maize bran, R-Rice bran, IA-Tropical white morning glory powder, WB-Wheat bran, HN-American hog-peanut powder and GW-Glycine powder. Means followed by the same letter within a column are not significantly different at p < 0.05.

Cricket farming yield is an important criterion for evaluating feed performance, particularly the impact of feed on cricket development time. The time required for the nymphs to develop into adults differed significantly across single plant products and blended reference feeds (F_11, 3588_ = 1179, p < 0.001) (**Fig 3**). The shortest average development period, 29.2 ± 0.2 days, was recorded with gallant soldier compared to the reference feed (28.9± 0.2) days. The longest developmental time was associated with rice bran (41.9 ± 0.2) days. Female *G*. *madagascarensis* reached the adult stage earlier than their male counterparts (F_1, 3588_ = 9.578, p < 0.001) (**S2 Fig in S1 File**). There was a significant difference in the development time of male and female crickets offered different single plant product feeds and the blended reference feed (F_1, 1490_ = 77.67, p < 0.001 for males, F_1, 2098_ = 128.00, p = 0.006 for females) (**S2 Fig in S1 File**).

#### 3.4.2 Effects of weeds, agro by-products powders and reference feed on the survival rate of *G*. *madagascarensis*

**Fig 4:**
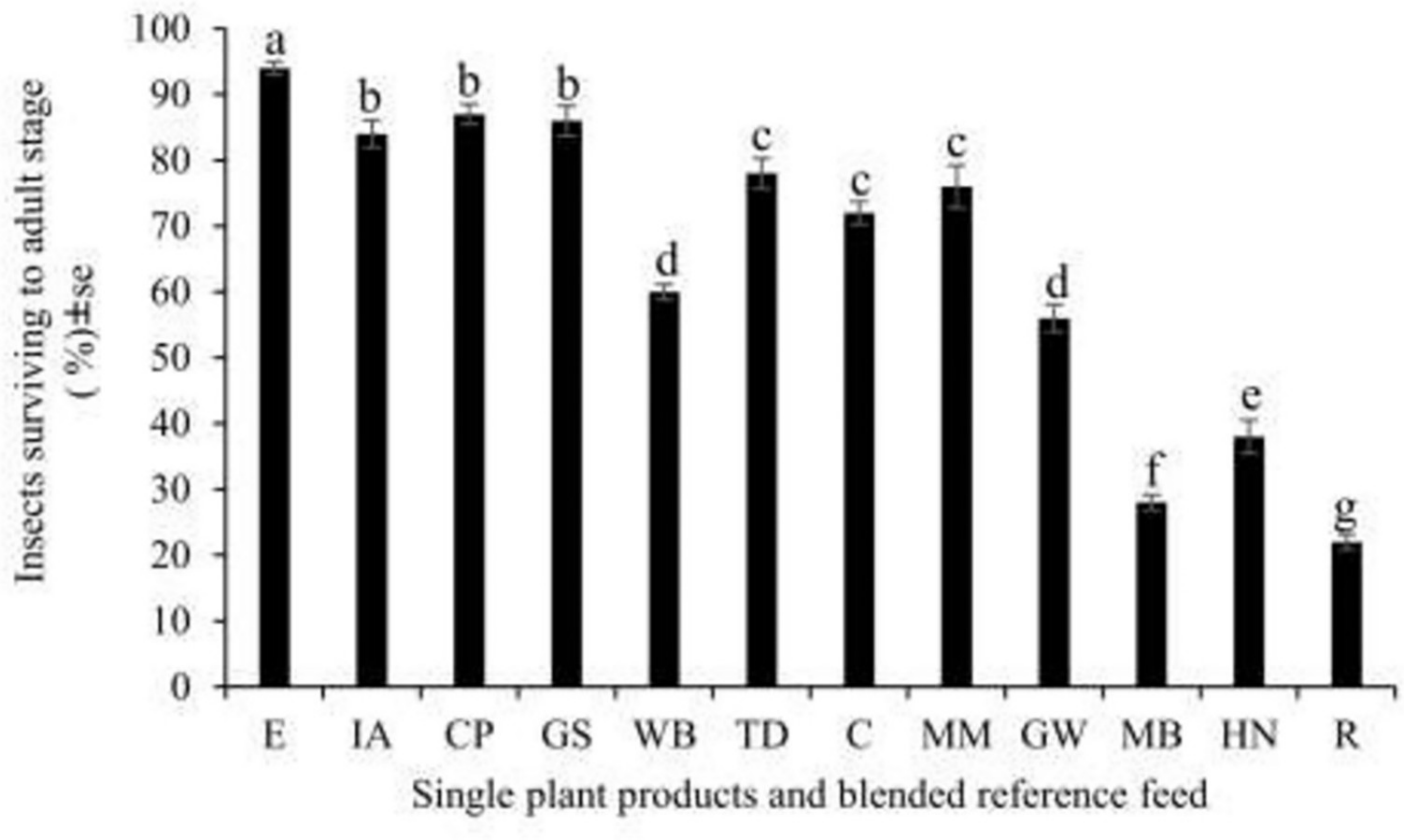
Mean (± se) proportion of crickets surviving to the adult stage as a function of the feed tested (n = 300). E-Blended reference feed, C-Cassava leaves powder, CP-Cowpeas powder, MM-Silverleaf *Desmodium* leaf powder, GS-Gallant soldier powder, MB-Maize bran, R-Rice bran, IA-Tropical white morning glory powder, WB-Wheat bran, HN-American hog-peanut powder and GW-Glycine powder. Means followed by the same letter within a column are not significantly different at p < 0.05.

Our data show a significant difference in the rate at which nymphs survive to the adult stage when fed on single plant products and reference feed (F_11, 24_ = 150.2, p < 0.001). The highest survivorship was observed in crickets fed on cow pea bran (87%) and the lowest survivorship was recorded for rice bran (22%) while the survivorship of crickets fed the reference feed was 94% (**Fig 4**).

#### 3.4.3 Effect of weeds, agro by-products powders and reference feed on wet body mass and body length of *G*. *madagascarensis*

**Fig 5:**
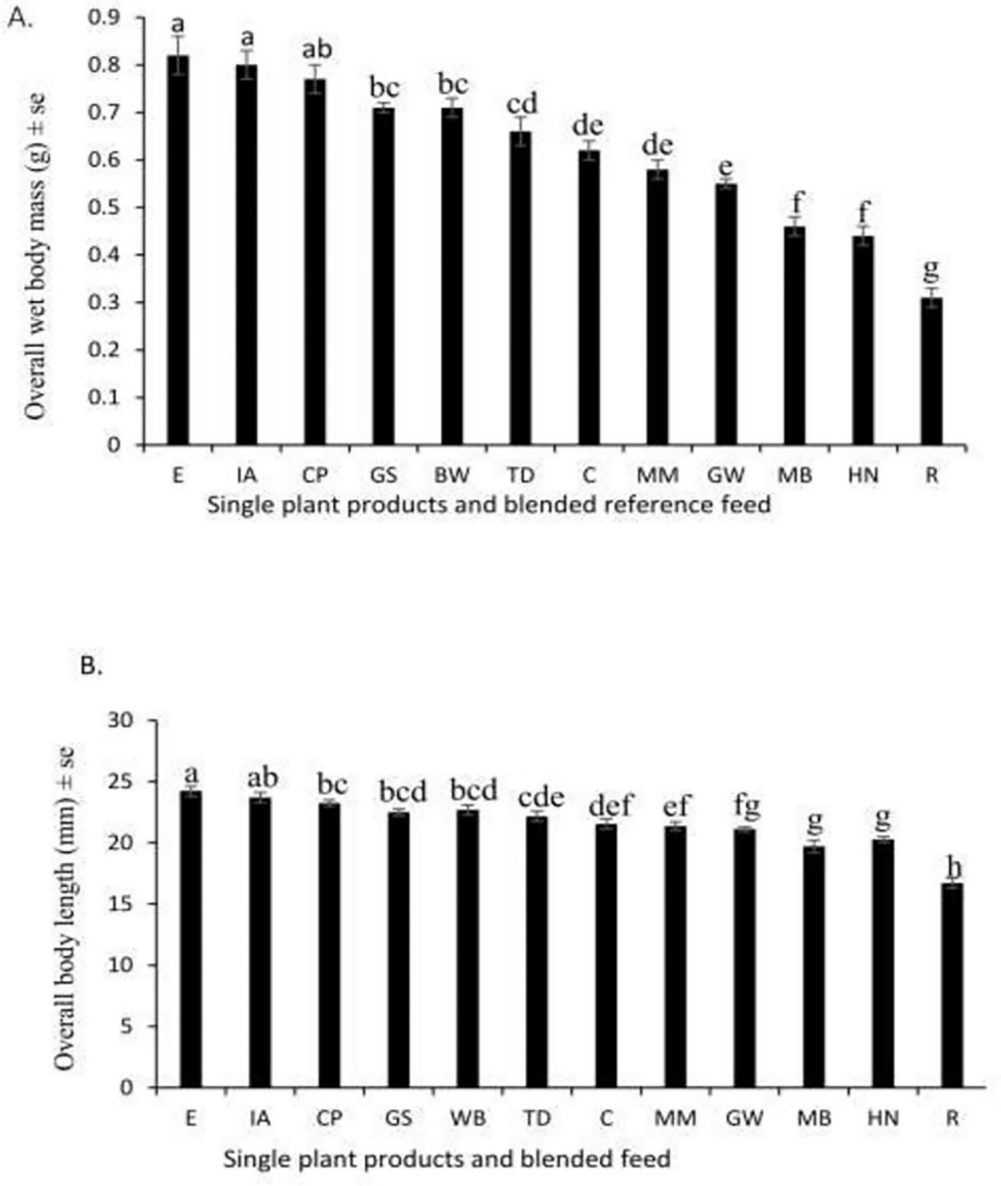
Mean (± se) wet body mass (A) and Mean (± se) body length (B) of adult *G*. *madagascarensis* reared on different single plant products and blended reference feed (n = 72). E- Blended reference feed, C-Cassava leaves powder, CP-Cowpeas powder, MM-Silverleaf *Desmodium* leaf powder, GS-Gallant soldier powder, MB-Maize bran, R-Rice bran, IA-Tropical white morning glory powder, WB-Wheat bran, HN-American hog-peanut powder and GW-Glycine powder. Means followed by the same letter within a column are not significantly different at p < 0.05.

Another parameter that impacts how feed affects farming yields is a diet’s influence on cricket mass and size. We therefore measured these parameters to assess candidate feed suitability. The heaviest and largest crickets were obtained using tropical white morning glory throughout the development period (**Fig 5A and 5B**). The adult wet mass varied significantly among crickets fed on different diets (F_11, 852_ = 63.32, p < 0.001). A significant interaction existed between sex and diet on body mass of *G*. *madagascarensis* (F_11, 852_ = 3.543, p < 0.001). Crickets fed tropical white morning glory had the highest average mass (0.80 g) compared to individuals fed the reference feed (0.82 g), while those fed rice bran had the lowest average mass (0.31 g) (F**ig 5A**). The wet body mass of female crickets was higher than that of male crickets (F_1, 852_ = 117.846, p < 0.001) (**S3 Fig in S1 File**). The body mass of male and female crickets varied among single plant products and the reference feed (F_11, 426_ = 31.27, p < 0.001 for males, F_11, 426_ = 51.97, p < 0.001) (**S3 Fig in S1 File**). The highest wet body mass in males and females was recorded in crickets fed tropical white morning glory feed (0.75 and 0.86 g) compared with the reference feed (0.73 and 0.91 g) (**S3 Fig in S1 File**). Similar observations were made for the adult body length of crickets reared on different single plant products and the reference feed (F_11, 852_ = 46.51, p < 0.001) (**Fig 5B**). There was also a significant interaction between sex and single plant products and the reference feed on body length (F_11, 852_ = 3.483, p < 0.001). Male crickets offered tropical morning glory had the longest bodies (23.3 mm) while males fed reference feed measured 23.4 mm and males fed on rice bran had the shortest bodies (15. 7 mm) (**S4 Fig in S1 File**). The wet body length of female crickets was longer than that of male crickets (F_1, 852_ = 117.846, p < 0.001) (**S4 Fig in S1 File**). The body length of males and females differed among single plant products and the reference feed (F_11, 426_ = 30.37, p < 0.001 for males, F_11, 426_ = 38.29, p < 0.001 for females). The longest female (24 mm) *G*. *madagascarensis* received either tropical white morning glory or chicken feed (25 mm) (**S4 Fig in S1 File**).

#### 3.4.4 Effect of weeds, agro by-products and blended reference feed on the reproductive parameters of *G*. *madagascarensis*

Reproductive parameters are important, particularly when growing a cricket colony. We, therefore, examined the impact of candidate feeds on four main reproductive parameters: the time it took for an adult female to start laying eggs (duration of pre-oviposition), female fecundity, egg hatchability and incubation time. Duration of pre-oviposition varied significantly with different single plant products and blended reference feed (F_11, 132_ = 19.59, p < 0.001). The pre-oviposition time was shortest in females raised on tropical white morning glory and blended reference feed (2 days), and the longest period was among those fed rice bran (~7 days) (**Table 3**). Fecundity was also significantly affected by the single plant products and blended reference feed (F_11, 132_ = 263.2, p < 0.001; **Table 3**). Fecundity correlated linearly with body weight (r = 0.87) and body length (r = 0.78). Interestingly, the different feeds tested had a strong impact on egg hatchability (F_11, 132_ = 114.9, p < 0.001). The highest hatchability was recorded for eggs laid by females fed with tropical white morning glory (93.42%) compared to the reference feed (92.92%), while the lowest hatchability was recorded for eggs from females raised on rice bran meal (53.42%) (**Table 3**). The feed used to raise the females also significantly impacted the incubation time of eggs (F_11, 1188_ = 198.00, p < 0.001). The shortest incubation time was observed in females fed with tropical white morning glory, cowpeas, wheat bran and gallant soldier (~7 days), a period similar to blended reference feed (7 days), while the longest was observed in females raised on rice bran (~10 days) (**Table 3**).

**Table 3:** The effects of different single plant products and blended reference feed on the reproductive parameters of *G*. *madagascarensis*.

Single plant products and blended reference feed	Preoviposition time (Days)	Fecundity (Eggs)	Egg incubation time (Days)	Egg hatchability (%)
E	2.25 ±0.25a	2335.83 ± 15.05a	7.22±0.04a	92.92±1.03a
IA	2.17 ±0.27a	2311.92 ±15.22a	7.05±0.07a	93.42±1.14a
CP	2.92 ±0.19b	2183.17 ± 55.01b	7.03±0.07a	89.67±1.28a
GS	3.08 ±0.23b	2042.75 ± 20.42c	7.15±0.06a	93.33±0.57a
WB	3.92 ±0.26bc	1961.17 ± 45.41cd	7.45±0.08b	92.00±0.44a
TD	3.25 ±0.28b	1943.58 ±39.06cd	8.36±0.05e	83.64±1.94b
C	4.75 ±0.35c	1931.67 ±18.04d	8.07±0.04d	88.50±1.14a
MM	4.83 ±0.41bc	1447.50 ± 40.67e	7.84±0.05c	81.75±0.94b
GW	5.25 ±0.35d	1431.14 ± 32.78e	9.03±0.10f	60.83±2.76c
MB	5.25 ±0.43d	1271.17 ± 3.34f	9.39±0.08g	60.42±1.91c
HN	5.67 ±0.40d	1044.67 ± 11.80g	9.04±0.08f	52.33±1.86d
R	6.67 ±0.40e	962.25 ± 5.21g	9.61±0.08h	53.42±1.93d

Note: E-Blended reference feed, C-Cassava leaves powder, CP-Cowpeas waste powder, MM-Silverleaf *Desmodium* leaf powder, GS-Gallant soldier powder, MB-Maize bran, R-Rice bran, IA-Tropical white morning glory powder, WB-Wheat bran, HN-American hog-peanut powder and GW-Glycine powder. Means are given ± se. Means followed by the same letter within a column are not significantly different at p < 0.05.

### 3.5 Cost of the selected weeds and agro by-products powders and blended reference feed, initial body mass, average body mass gain, feed consumed by individual cricket, feed conversion ratio, feed consumed by one kilogram of crickets, price of feeding one kilogram of live crickets

When selecting the best feed, yield indicators are not the only parameters to consider. The cost of feed will fundamentally affect the economic sustainability of farms both small and large. The prices of the feed tested, initial body mass, final body mass gain, average body mass gain, feed consumed by individual *G*. *madagascarensis*, feed conversion ratio, feed consumed by one kilogram of crickets, and price of feeding one kilogram of live crickets are given in **Table 4**. All single plant products tested were significantly cheaper than the blended reference feed (i.e. chicken feed). *Gryllus madagascarensis* fed on single plant products and reference showed significantly high final body mass gain (F_11, 274_ = 248005, p < 0.001). Cricket reared on both single plant product and reference feed had a highly significant feed intake (F_11, 1188_ = 891.0, p < 0.001). Daily feed intake per cricket raised on tropical white morning glory (0.043 gDM) which contributed to the heaviest crickets was comparable to the blended reference feed (0.045 gDM) (Table 3). The highest daily intake was recorded on crickets provided with cassava leaf tops powder (0.046 gDM) while the lowest daily intake was recorded on rice bran (0.026 gDM). Crickets raised on single plant products and reference feed had a high significant average daily gain (ADG) (F_11, 1188_ = 967.0, p < 0.001). On the other hand, crickets fed on tropical white morning glory and cowpea bran showed the highest average daily gain (0.026 g). The lowest average daily gain (0.007g) was detected on crickets provided rice bran. Crickets given single plant products and reference feed demonstrated a highly significant feed conversion ratio (FCR) (F_11, 1188_ = 93118, p < 0.001). The crickets raised on tropical white morning glory powder and cowpea had the lowest FCR (1.65 and 1.69 respectively) compared to the reference feed (1.61) and other single plant products. The single plant products and reference feed used to produce one kilogram of live crickets varied significantly across the feeds. The cost of feeding per kg live mass gain of crickets (in Malagasy ariary) differed across the single plant products and the reference. The cheapest mean cost per kg live mass was observed in crickets fed feed tropical white morning glory powder, Silverleaf *Desmodium* powder, Gallant soldier powder, taro leaf powder, American hog-peanut powder and glycine powder (0 Malagasy ariary) and the expensive mean cost per kg live mass was recorded in crickets fed feed blended reference feed (~209 Malagasy ariary). Crickets reared on tropical white morning glory powder resulted in the lowest number of crickets making up one kilogram of live weight comparable to the reference feed while crickets fed on rice resulted in the highest number of crickets required to make one kilogram of live weight.

**Table 4:** Cost of the selected weeds and agro by-products powders and blended reference feed, initial body mass, final body mass gain, average body mass gain, feed consumed by individual cricket, feed conversion ratio, feed consumed by one kilogram of crickets, price of feeding one kilogram of live crickets.

Single plant products and blended reference feed	Cost of single plant product and reference feed kg (Malagasy ariary)	Cost per Kg (USD)	Mean initial body mass (g)	Mean final body mass gain (n = 24)	Average daily body mass gain (g) (n = 100)	Mean daily feed intake (g) (n = 100)	Feed conversion ratio (n = 100)	Number of crickets per kilogram live body mass	Quantity of feed consumed per kilogram cricket live body mass (gDM)	Cost of producing one kg of live crickets (Malagasy ariary)
Blended reference feed	3800	0.84	0.001± 0.000	0.819±0.01a	0.028±0.001a	0.045±0.001b	1.61±0.01a	1250	54.88	208.54
Cassava leaf tops powder	1000	0.22	0.001±0.000	0.619±0.01f	0.021±0.001d	0.046±0.001a	2.19±0.01f	1613	74.20	74.20
Cowpeas bran	1000	0.22	0.001±0.000	0.769±0.01c	0.026±0.001b	0.044±0.001c	1.69±0.01c	1299	57.16	57.16
Silverleaf *Desmodium* powder	0	0	0.001±0.000	0.579±0.01g	0.017±0.001e	0.039±0.001f	2.29±0.01h	1725	67.28	0
Gallant soldier powder	0	0	0.001± 0.000	0.709±0.01d	0.024±0.001c	0.041±0.001e	1.71±0.01d	1409	57.77	0
Maize bran	800	0.18	0.001± 0.000	0.459±0.01i	0.012±0.001g	0.039±0.001f	3.25±0.01j	2174	84.79	67.83
Rice bran	1000	0.22	0.001± 0.000	0.309±0.01k	0.007±0.001i	0.026±0.001h	3.71±0.01l	3226	83.88	83.88
Taro leaf powder	1000	0.22	0.001± 0.000	0.659±0.01e	0.021±0.001d	0.045±0.001b	2.14±0.01e	1516	68.22	68.22
Tropical white morning glory powder	0	0	0.001± 0.000	0.799±0.01b	0.026±0.001b	0.043±0.001d	1.65±0.01b	1250	53.75	0
Wheat bran	1000	0.22	0.001± 0.000	0.709±0.01d	0.020±0.001c	0.030±0.001g	2.25±0.01g	1409	42.27	42.27
American hog-peanut powder	0	0	0.001± 0.000	0.439±0.01j	0.011±0.001h	0.039±0.001f	3.55±0.01k	2273	88.65	0
Glycine powder	0	0	0.001± 0.00	0.549±0.01h	0.016±0.001f	0.039±0.001f	2.44±0.01i	1819	70.94	0

Note: 1 USD (US dollar) = 4558 MGA during the period of study (2023/2024). Where applicable means (± se) are provided.

Zero (0) value means the weed or agro by-product was not purchased.

## 4 Discussion

The present study aimed to identify weeds and agricultural by-products available in central Madagascar and assess their suitability as feed for breeding the cricket *G*. *madagascarensis* based on their nutritional content, impact on cricket size as a determinant of yield, development, reproduction, and cost. Forty-eight weeds and 18 agro by-products were identified to the species level. Weeds and agro by-products were then dried and ground into single plant products. We tested the acceptability of all 66 of the single plant products. The results of the feeding challenge showed that the cricket accepted all 66 single plant products at varying consumption degrees. The most accepted single weed product was African white morning glory, while cassava leaf powder was the most accepted single agro by-product.

The variation in acceptance recorded among different single plant products in the present study could be attributed to lower nutrient concentration which could have led to increased consumption to meet the nutritional requirements of the cricket [66]. Other components such as antinutrient components are known to influence rates of acceptability by insects and antinutrients differ considerably across species of single plant products [67–70], however, this was not tested in the current study.

Nutritionally, the top 11 accepted single plant products proved to be excellent sources of nutrients required by the crickets for their normal development, growth, survival and reproduction. These plant products exhibited varying levels of protein, carbohydrate, phosphorus, iron, copper and calcium, and lower levels of fats, sodium and manganese. The shortest development time was associated with gallant soldier, which had a protein content comparable to that of blended reference feed. The highest survival rate, which was comparable to the blended reference diet, was recorded with cow pea bran, tropical white morning glory and gallant soldier. The largest body length and weight, the shortest preoviposition duration and the highest female fecundity were also recorded on crickets fed on tropical white morning glory. Together with wheat bran and gallant soldier, these feeds also allowed the shortest egg hatching time. Depending on the parameter considered, the best breeding performances were therefore observed with chicken feed, gallant soldier, tropical white morning glory and cassava leaves tops, wheat bran and cowpea bran. Possible factors influencing the breeding performance of the tested feed are discussed below.

In the current study, *G*. *madagascarensis* reared on the reference feed took approximately 29 days from juveniles to the emergence of adult crickets compared to an early study of 70 days on *G*. *madagascarensis* reared on chicken feed with 20% protein [71]. The difference between the current reference and previous reference feeds could be a result of differences in nutrition content and other factors such as temperature and relative humidity under which the studies were conducted. The present study has demonstrated that *G*. *madagascarensis* was able to complete its juvenile cycle when fed with the top 11 accepted feeds (six most accepted weeds and five agro by-products) and the blended reference feed. The variation in development time could be attributed to the differences in the nutrition content of the single plant products and reference feed. Development of *G*. *madagascarensis* juveniles to adult stage in this study was shorter (29–42 days) than the development time of juveniles of *Acheta domesticus* (42–49 days) [70], *Gryllus assimilis* (44–64 days) [72], *Gryllodes sigillatus* (34–60 days) [73], *Gryllus bimaculatus* (36–48) [44]. and *Scapsipedus icipe* (65–107 days) [45]. fed on different single plant products and blended reference feed.

Higher protein quantities did not drastically shorten the duration of development. Previous studies have reported that speeding development in crickets requires a diet featuring more carbohydrates and less fat [74,75]. In our case, gallant soldier, with 52% carbohydrates and 3% fat, led to the shortest development time comparable to that of the blended reference feed, which has a carbohydrate content of 57% and 4% fat. By contrast, rice bran, associated with the longest development time, has the highest levels of carbohydrates (63%) and high crude fat content (6%). This suggests that for faster development, the crickets require their feed to have an optimal ratio of protein to carbohydrate to fat. Protein is required in the diet since the amino acids they contain are used to synthetize cricket proteins [61,76]. On the other hand, carbohydrates and fats provide the necessary energy, particularly during molting, searching for food and water, mating and other metabolic activities. Interestingly, despite their higher protein content, some other single plant products tested (including tropical white morning glory, cowpea meal, cassava leaf tops, wheat bran, taro leaf meal and glycine) resulted in a longer developmental time than gallant soldier and the reference feed. This could be because feeding crickets on single plant products with high crude fiber led to high bulking of their digestive system which resulted in less nutrient absorption and less ratio of dietary protein to energy compared to the reference feed.

The current study showed that variations occurred in the survival rate of *G*. *madagascarensis* when fed on different single plant products and reference feeds. This fact could be attributed to differences in nutritional value among the different feeds [66]. Previous studies have demonstrated that feeding newly emerged cricket nymphs on single plant products rich in nutrients such as tropical white morning glory, cowpeas bran, gallant soldier, silver leaf *Desmodium*, taro meal and cassava leaves leads to reduced mortality [24]. On the other hand, rearing crickets on low-quality single plant products such as rice bran in our case led to an increased nymph mortality as a result of unrealized nutritional demands [54,77,78]. In the current studythe survival rate of *G*. *madagascarensis* nymphs fed on powders of tropical white morning glory, cowpea bran, gallant soldier, silver leaf *Desmodium*, taro meal and cassava, which had more proteins and carbohydrate, than the blended reference feed, was somewhat lower. This could be possibly because of high levels of fiber in single plant products which reduced the bioavailability of the respective nutrients to the crickets unlike in the reference feed. The level of protein in a feed is important in determining the survival rate in crickets. Our study showed a higher survival rate in *G*. *madagascarensis* reared on single plant products and reference with a protein content of over 20%. The current study differs from the study of [61]. which indicated no further enhancement in survival of *G*. *bimaculatus* reared on diets with protein content beyond 17%. However too much or too low protein in diets has been reported to increase mortality in insects such as ants [78]. Therefore, it is recommended that a farmer should not feed crickets with feeds having too much since the protein will be toxic to the cells of the cricket while too low protein will lead to cannibalism in crickets. On the other hand, previous reports have shown that for farmed crickets to attain high survivorship, they need to be reared on optimal quantity of sodium (2.1 g/kg DM), iron (644 mg/kg DM), phosphorus (9.9 g/kg DM), calcium (90.7 g/kg DM), potassium (10.2g/kg) zinc (199 mg/kg DM), magnesium (3.2 g/kg DM), copper (23 mg/kg DM) and manganese (293 mg/kg DM) [79–81]. The current study has demonstrated that the single plant products fed to *G*. *madagascarensis* had a lower level of the recommended mineral elements for crickets. Despite the lower minerals content in our study, *G*. *madagascarensis* had higher survival rates when fed on some single plant products comparable to that of the reference feed However, for the farmers to realize the highest survival rates in *G*. *madagascarensis* than the one reported here, they can blend the single plant by-product in different proportion or supplement them with the respective minerals to increase the mineral content to the optimum level. Therefore, while preparing the cricket feed recipes, the ingredients should be mixed in a manner that results in optimal nutrient levels for the crickets. The single plant products used in this study, which are homogeneous feeds, seem to have had less optimal quantities of nutrients for cricket survival when compared to the blended reference feed which is heterogeneous.

The findings indicate that feed is known to greatly influence the body length and body mass of crickets [14,44]. Determining the right feed with optimum macro- and micronutrients to promote longer, heavier bodies in *G*. *madagascarensis* is, therefore, integral to increasing yield and profits and attaining sustainability. Our study showed that crickets fed on different single plant products with varying nutrients attained different body lengths and body mass. Large body weight and length could be a result of high levels of nutrients such as protein, carbohydrates and fat, calcium, sodium, manganese and iron in the corresponding feed [14]. The current findings agree with previous studies, which indicated that crickets such as *Gryllus bimaculatus* and *Acheta domesticus* fed by-products rich in protein, carbohydrate and fat produced heavier and longer crickets compared to those fed protein-rich/low carbohydrate diets [14,61]. Crickets fed rice bran with very high carbohydrates and fats displayed reduced size and weight [77] observed that crickets fed on diets with high or too-low-fat feed for a long period had reduced body weight due to oxidative stress and were unable to compensate for inadequate nutrients. This study concurs with previous studies which showed that as generalist feeders, crickets can ingest, digest and assimilate nutrients from a wide variety of single plant products and still complete normal growth and development [48,61]. This implies that *G*. *madagascarensis* can be reared on different single plant products found locally in every region of Madagascar, and more generally that our results on *G*. *madagascarensis* may also apply to other cricket species, including in mainland sub-Saharan Africa, where the same single plant products are also widespread.

The current study showed that different types of feed affected preoviposition duration, fecundity, egg incubation period and egg hatchability. The shortest preoviposition duration was recorded when females were fed on tropical white morning glory and reference feed, while the longest duration was recorded on females fed with rice bran. This could be due to that the protein content in the tropical white morning glory and the reference diet were readily available to the cricket. The fecundity of *G*. *madagascarensis* was highest on tropical white morning glory which is comparable to the one of the reference feed, which contained higher protein, and the lowest fecundity was associated with the rice bran diet. Previous studies have indicated that variations in preoviposition, fecundity, egg incubation period and egg hatchability among weeds and agro by-products depend on the quantity of protein, carbohydrate, and mineral elements in the feeds [51,82]. The level of nutrients in feeds offered to the crickets affected reproductive activities preceding egg production parameters. For instance, studies on *Gryllus assimilis* and *Gryllodes sigillatus* had demonstrated that the amount of protein (over 20–25%) and carbohydrate ranging between 54.50 to 58.00% in diets influences mating through male calling behaviour and egg laying. These activities demand significant energy that can only be obtained from metabolizing proteins and carbohydrate [83]. This finding is in agreement with the current study which shows that *G*. *madagascarensis* fed on African tropical morning glory (23.45% protein) and chicken feed (20.03%) with carbohydrate range of 54.8 to 57.31% had higher fecundity compared to other feeds with low or higher carbohydrate. A gravid cricket will lay far more eggs than a poorly mated cricket [76]. It is reported that crickets fed on diets rich in high-quality protein, carbohydrate, sterol, iron and phosphorus will have larger, faster-maturing ovaries able to accommodate more eggs than crickets fed on low-quality diets [76]. This result is similar to the study conducted on the effect of high levels of proteins, carbohydrate and sterol on fecundity in other insects feeding on host plants; fecundity was reported to increase as the content of these elements rose [78,84,85]. The fecundity of *G*. *madagascarensis* was also discovered to depend on the cricket’s body size and weight. For instance, when the correlation of body length, body weight and fecundity was assessed, the results indicated that the fecundity of *G*. *madagascarensis* increased linearly with wet body length and body weight, following early reports for other cricket species [45,48]. It is worth noting that the fecundity of *G*. *madagascarensis*, which produces 962.3–2338.8 eggs over a lifetime, is in the same range as that of other crickets such as *G*. *bimaculatus*, *Scapsipedus icipe*, *G*. *assimilis* and *Acheta domesticus* reared on weeds and agro by-products [59,86–89].

In terms of feed cost, the current study shows that the single plant products obtained from weeds and agro by-products are cheaper than the reference diet. Therefore, single plant products offer an economic advantage while being able to fulfil the dietary needs of *G*. *madagascarensis*. Though single plant products differ widely in their nutritional content, in general, they offer sufficient nutrients for crickets [66]. The current global search for cheap feed suitable for edible crickets has been triggered by the exorbitant cost of chicken feed, which discourages many people from farming crickets [43,90]. We therefore recommend that cricket farmers in Madagascar, a country with a high diversity of weeds and agro by-products, make use of locally available single plant products as feed for their crickets. Since supplies of single plant products have seasonal limitations, cricket farmers are advised to harvest them when they are in season, rinse them with clean water, sun dry the harvest, and process them into powders before storing them for future use. Wide adoption of this method can be encouraged with awareness campaigns among cricket farmers.

## 5. Conclusion

The current study demonstrates that *G*. *madagascarensis* is a polyphagous cricket that consume a variety of single plant products and reference feed at different rates as reflected in its acceptance of 48 weeds and 18 agro by-product. Moreover, this study has identified the 11 most accepted single plant products which are locally available and cheap for sustainable farming of edible crickets in Madagascar. The findings of this study shows that *G*. *madagascarensis* can develop, grow, survive and reproduce when reared on different single plant products as compared to the reference feed. The studied single plant products contain the required nutrient content, albeit in different quantities, to sustain the development, growth, survival and reproductive fitness of farmed crickets. However, since no single plant product can provide all the required nutrients in the right amount to support proper development, growth, survival and reproduction in farmed crickets, farmers are recommended to develop blended feeds which match the blended reference feed. It is, therefore, anticipated that the blended feeds to be developed will then influence *G*. *madagascarensis* traits such as development time, survivorship, body weight, body length and female reproductive parameters, leading to a significant impact on the population structure, yield, and overall success of farming edible crickets. Given that this is the first study of its kind conducted on *G*. *madagascarensis*, the findings offer groundbreaking information that may be useful when designing feeds for farming *G*. *madagascarensis* in Madagascar and other parts of the world. These findings further contribute to the body of knowledge needed to rear crickets on a large scale to mitigate global food insecurity and malnutrition. Further studies are needed to develop optimized blended feeds combining different proportions of various single products from weeds and agro by-products to facilitate maximum development, survival, growth and reproduction during rearing. This will reduce farmers’ reliance on chicken feed to support their crickets. Lastly, the use of weeds and agro by-products could improve the environmental sustainability of cricket feeds and cricket farming, and promote a circular economy.

## Supporting information

S1 File (DOCX)

S1 Data (XLSX)
