# Supplementary material for: Weeds and agro by-products for sustainable farming of edible field cricket, Gryllus madagascarensis (Orthoptera: Gryllidae)
Source: PLoS One. 2025 Jan 3;20(1):e0313083. doi: 10.1371/journal.pone.0313083 (PMC11698383; doi:10.1371/journal.pone.0313083)
Supplement: S1 File — (DOCX) [file pone.0313083.s001.DOCX]

Supporting information

**Weeds and agro by-products for sustainable farming of edible field cricket, *Gryllus madagascarensis* (Orthoptera: Gryllidae)**

**Henlay J.O. Magara^1*^, Cédrique L. Solofondranohatra^1^**, **Sylvain Hugel^1, 2^, Brian L. Fisher^1, 3^**

^1^ Department of Feed Development, Madagascar Biodiversity Center, Antananarivo, Madagascar

^2^ Institut des Neurosciences Cellulaires et Intégratives, UPR 3212 CNRS-Université de Strasbourg, Strasbourg, France.

^3^ Department of Entomology, California Academy of Sciences, San Francisco, CA, United States.

*** Correspondence:** H.J.O. Magara, **Tel.:** + 254-721496376 Email: mhenlay@gmail. com

## Supporting Figures


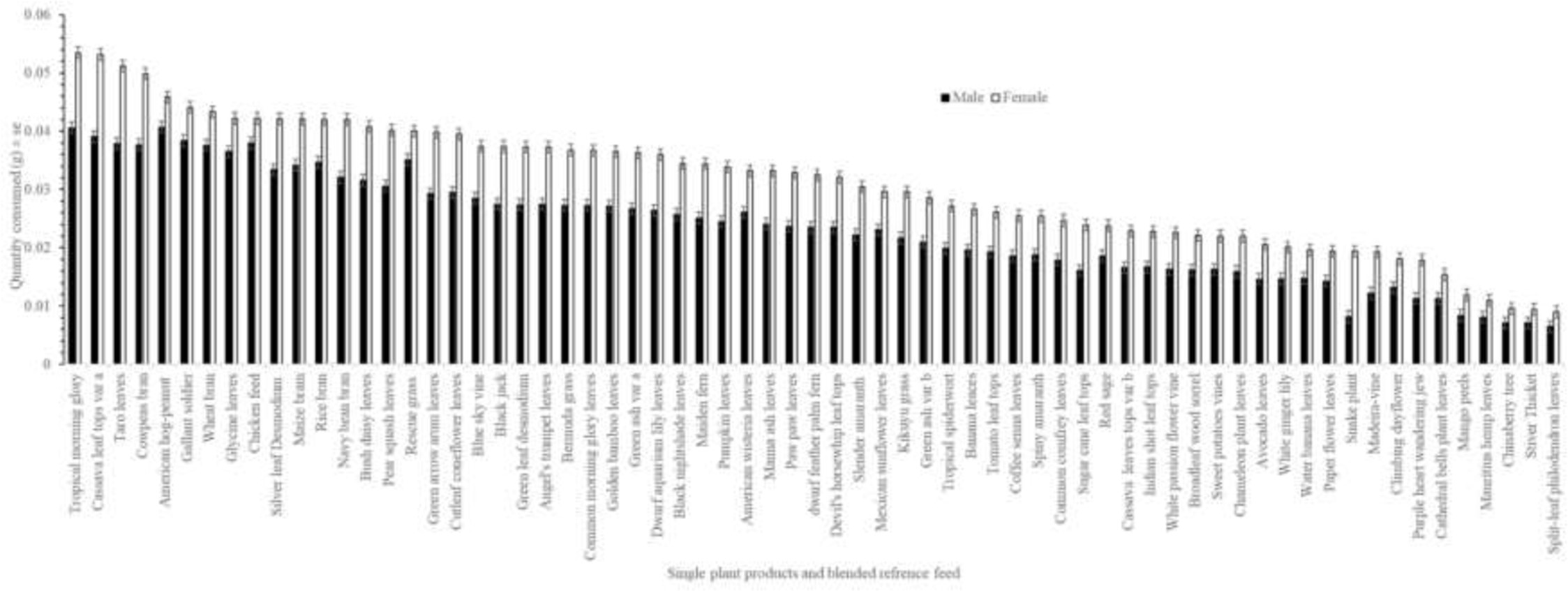


**S1 Fig.** Acceptance of the 66 single plant products and blended reference feed by respective based on a no-choice test (n=30). The histogram shows the mean ± se quantity of single plant products compared to the reference diet, blended reference feed consumed by *G. madagascarensis* adult males and females in 24 hours. The tested feeds are ordered by the amount consumed.


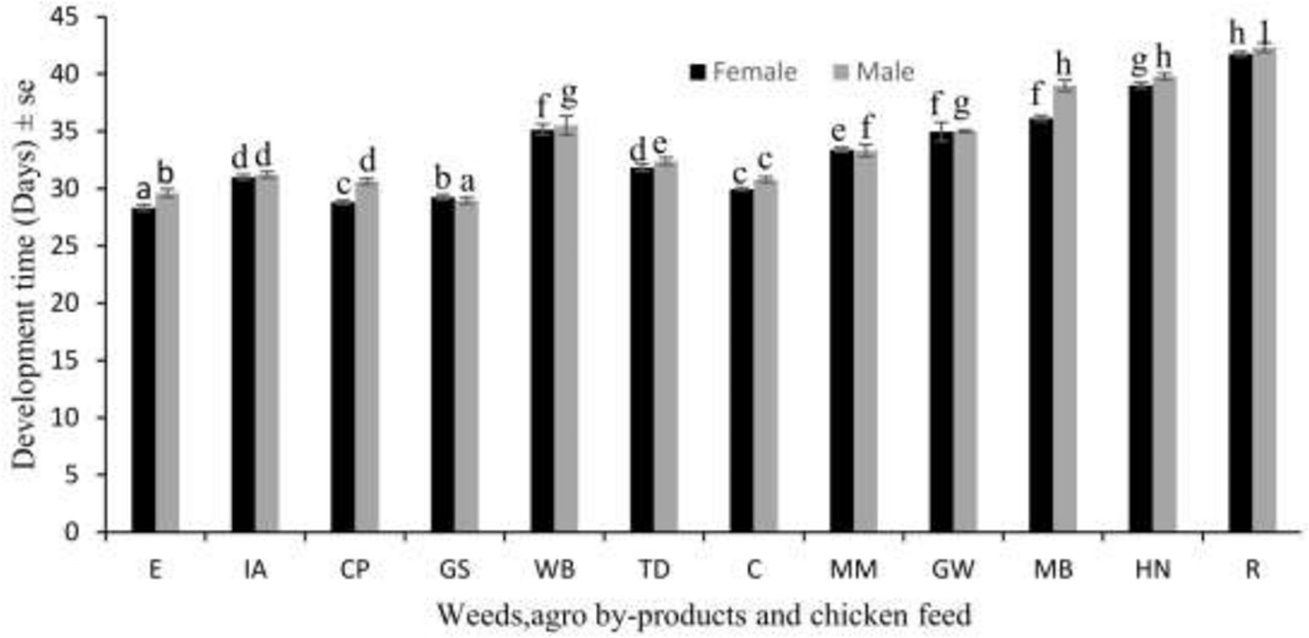


**S2 Fig.** Mean (± se) development time of males and females of *G. madagascarensis* reared on different weeds, agro by-products, and chicken feed (n=300). E- Blended reference feed, C-Cassava leaves powder, CP-Cowpeas powder, MM-Silverleaf *Desmodium* leaf powder, GS-Gallant soldier powder, MB-Maize bran, R-Rice bran, IA-Tropical white morning glory powder, WB-Wheat bran, HN-American hog-peanut powder and GW-Glycine powder. Means followed by the same letter within a column are not significantly different at p < 0.05.

A


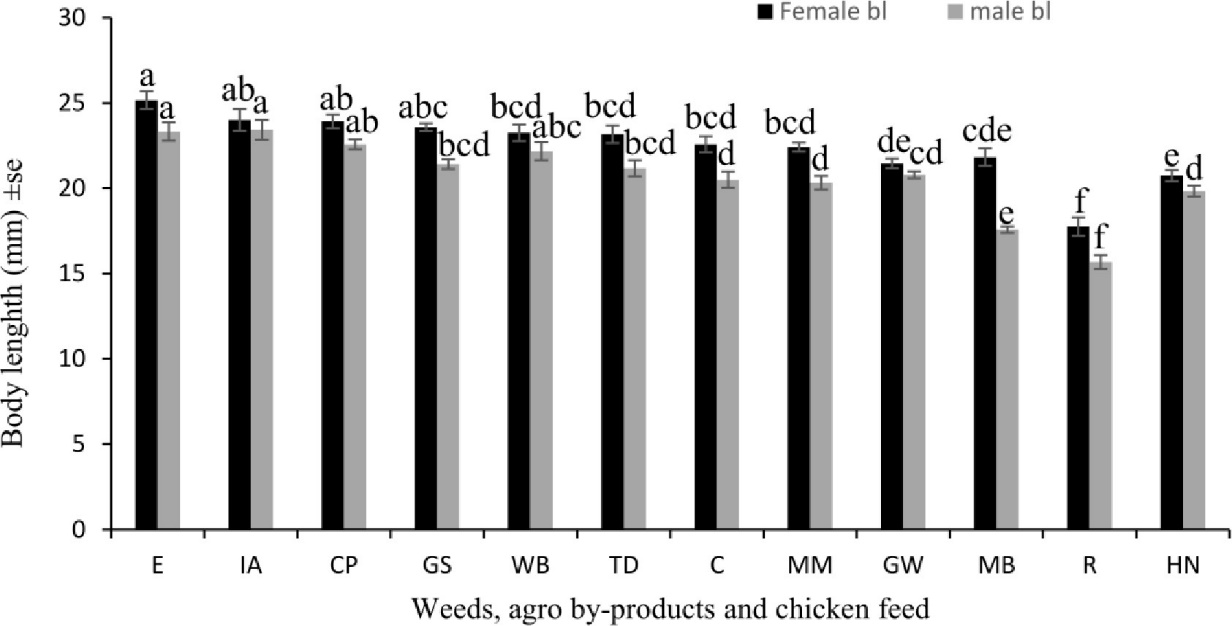


B


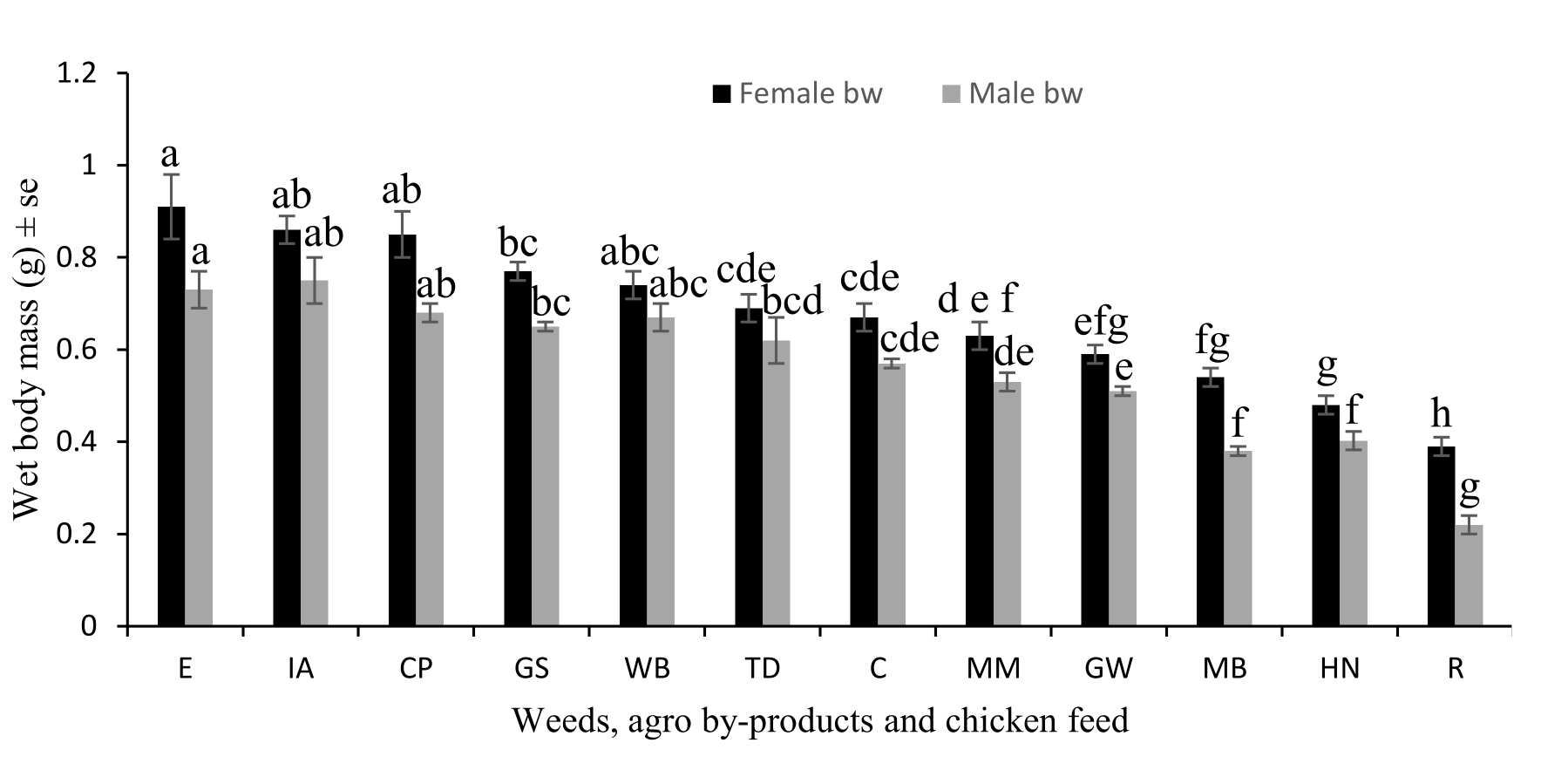


**S3 Fig.** Mean (± se) wet body mass (A) and Mean (± se) body length (B) of adult males and females of *G. madagascarensis* reared on different single plant products and blended reference feed (n=72). E- Blended reference feed, C-Cassava leaves powder, CP-Cowpeas powder, MM-Silverleaf *Desmodium* leaf powder, GS-Gallant soldier powder, MB-Maize bran, R-Rice bran, IA-Tropical white morning glory powder, WB-Wheat bran, HN-American hog-peanut powder and GW-Glycine powder. Means followed by the same letter within a column are not significantly different at p < 0.05.
